# Metagenomic analysis of the effects of plant- and yeast-based formulations on the grapevine leaf microbiome of cv. ‘Touriga Franca’

**DOI:** 10.3389/fpls.2025.1637143

**Published:** 2025-08-14

**Authors:** Eliana Monteiro, Paula Baptista, Sofia Silva, Márcia Carvalho, Radek Bragança, Kieran J. Guinan, Neerakkal Sujeeth, Isabel Cortez, Berta Gonçalves, Isaura Castro

**Affiliations:** ^1^ Centre for the Research and Technology of Agro-Environmental and Biological Sciences (CITAB), University of Trás-os-Montes e Alto Douro (UTAD), Vila Real, Portugal; ^2^ Institute for Innovation, Capacity Building and Sustainability of Agri-food Production (Inov4Agro) (UTAD), Vila Real, Portugal; ^3^ Mountain Research Center (CIMO), Polytechnic Institute of Bragança, Bragança, Portugal; ^4^ Department of Genetics and Biotechnology, University of Trás-os-Montes e Alto Douro (UTAD), Vila Real, Portugal; ^5^ BioComposites Centre, Bangor University, Bangor, United Kingdom; ^6^ Clash Industrial Estate, BioAtlantis Ltd., Tralee, Ireland; ^7^ Department of Agronomy, University of Trás-os-Montes e Alto Douro (UTAD), Vila Real, Portugal; ^8^ Department of Biology and Environment, University of Trás-os-Montes e Alto Douro (UTAD), Vila Real, Portugal

**Keywords:** leaves microbiome, microbial community diversity, metabarcoding, plant extracts, Vitis vinifera L., yeast-based formulations

## Abstract

**Introduction:**

Grapevine is highly susceptible to fungal diseases such as downy mildew and powdery mildew, which are traditionally managed through the intensive use of chemical fungicides. However, in the context of increasingly sustainable viticulture, biofungicides derived from plant and yeast extracts are gaining attention. Despite this, their impact on the grapevine leaf microbiome, crucial for plant health and disease resilience, remains underexplored.

**Material and methods:**

This study evaluated the effects of foliar applications of biofungicides (nettle extract, Japanese knotweed extract, and a yeast-based formulation - T66 and T90) in comparison with conventional chemical treatments and control (no treatment). Over two consecutive growing seasons, high-throughput sequencing was used to assess the diversity and composition of fungal and bacterial communities on grapevine leaves.

**Results:**

Bacterial communities were more sensitive to treatments and interannual variability than fungal communities, which remained relatively stable. Conventional treatment (CT) showed the highest influence on fungal and bacterial composition, reducing the diversity of both. Some important fungal (*Aureobasidium* and *Sporobolomyces*) and bacterial (*Pseudomonas* and *Sphingomonas*) genera associated with the promotion of plant growth, health, and biocontrol were detected.

**Discussion:**

These findings reinforce the potential of new treatments with putative fungicide effects to modulate the leaf microbiome, particularly bacterial communities, without disrupting the natural fungal balance. Thus highlight their relevance for developing sustainable viticultural practices aimed at improving plant protection.

## 1 Introduction

There is a growing awareness of the environmental and health impacts of indiscriminate chemical pesticide use in agriculture. In line with the Green Deal’s “Farm to Fork” strategy, the European Commission aims for ‘at least 50% of the EU’s agricultural land to be under organic farming by 2030’. It is expected that stricter controls on certain fungicides will lead to their potential withdrawal from the market in the near future. Challenges are also being faced in controlling certain fungal diseases, which may require more frequent spraying and repeated usage of the same active compound, potentially contributing to the emergence of pathogen resistance (Koledenkova et al., 2022; Monteiro et al., 2022). Furthermore, climate change characterized by dry and hot periods followed by unexpected rainfall, affects grapevine growth cycles and extends the window of susceptibility to fungal diseases, making its control more challenging particularly as they can develop more quickly and aggressively (Elad and Pertot, 2014; Monteiro et al., 2022).

The Demarcated Douro Region (DDR) is the largest and most heterogeneous mountainous wine region in the world and known for its unique terroir and Mediterranean climate (Fraga et al., 2017). Downy mildew (caused by *Plasmopara viticola* (Berk. & M.A. Curtis) Berl. & De Toni) and powdery mildew (caused by *Erysiphe necator* (Schwein.)), are obligate biotrophic pathogens and major contributors to some of the most problematic diseases in grape production (Karthick et al., 2019; Koledenkova et al., 2022). These diseases cause both quantitative and qualitative losses, affecting grape clusters (reducing harvest yields) and leaves (Karthick et al., 2019; Koledenkova et al., 2022). The increased difficulty in controlling these diseases combined with the need to maintain high productivity, has led farmers to adopt intensive crop protection practices resulting in high production costs (Ortega et al., 2023). In response, there is a growing need for sustainable farming systems that reduce chemical pesticides use. Plant- and yeast-based formulations offer promising alternatives. For instance, nettle extracts are rich in compounds such as saponins, flavonoids, tannins, proteins, and amino acids (Joshi et al., 2014), and Japanese knotweed extracts contain bioactive compounds like resveratrol and emodin (Oleszek et al., 2019; Borovaya et al., 2020). Both plant extracts demonstrated potential to improve the physiological and biochemical parameters of the grapevine (Monteiro et al., 2024a, 2024b) and presented antimicrobial effects (Rodino et al., 2018; Ghazal et al., 2019). Yeasts, commonly used in various industries, also exhibit plant-protective properties, including the production of volatile compounds, toxins, lytic enzymes, and the induction of plant resistance pathways (Puccioni et al., 2025). Studies have demonstrated their ability to enhance grape and wine quality and control downy mildew (Garde-Cerdán et al., 2017; Puccioni et al., 2025).

Despite these advances, the effect of biofungicides on the plant microbiome particularly in grapevines remains underexplored. The interactions between plants and their associated microbiomes, whether in the rhizosphere or phyllosphere, play a critical role in plant health, productivity, and stress resilience (Müller et al., 2016; Colla et al., 2017). Understanding the microbial community composition can provide valuable insights for managing disease pressure and improving grape quality (Leveau and Tech, 2011). High-throughput sequencing technologies have enabled a better understanding of the grapevine microbiome, revealing insights into both above- and below-ground microbial communities (Wei et al., 2018; Fadiji and Babalola, 2020).

Given the global importance of viticulture, and particularly in the DDR, this study aimed to investigate the effects of foliar applications of biofungicides derived from plant and yeast extracts in comparison with conventional chemical treatments. Over two consecutive years, we assessed their impact on the diversity and composition of fungal and bacterial communities on grapevine leaves, using metagenomic tools to analyze the grapevine leaf microbiome, which plays a key role in both grape production and wine quality.

## 2 Materials and methods

### 2.1 Plant material and sampling

Samples were obtained from the black skinned *Vitis vinifera* cv. ‘Touriga Franca’, grafted on 1103P rootstock, in two growing seasons (2020 and 2021). The trial was installed in an experimental vineyard located at University of Trás-os-Montes e Alto Douro (41°17’14.8”N 7°44’14.8”W, 500m above sea level), *Baixo Corgo* sub-region of the Douro Demarcated Region, Vila Real, northern Portugal. The topsoil (0–20 cm) exhibited a medium texture, 2.24% of organic matter, 47 mg/kg of P_2_O_5_, 104 mg/kg of K_2_O and a pH (KCl) 5.5. Vines were managed in rainfed conditions and grown using standard cultural practices commonly employed by commercial farmers. Vines were trained to bilateral Guyot and pruned to 12 buds per plant and spaced 2.20 m × 1 m between and along the rows. This area has Mediterranean climatic characteristics, with a warm-temperate climate with dry and hot summers, and higher precipitation during the winter months and very low during the summer. Monthly temperature and precipitation values were recorded by a weather station located near to the experimental site and are shown in **Supplementary Figure 1**.

Three replicates of six plants per treatment randomized along four vineyard rows of 36 plants were sprayed between leaves unfolded (BBCH 11) and veraison (BBCH 81) (Lorenz et al., 1995). Foliar sprayings were conducted during the morning, covering the whole canopy and according to disease incidence and weather conditions. Six different foliar treatments were tested, namely: i) nettle extract - NE (*Urtica* sp.; Bangor University) (3%); ii) Japanese knotweed extract - JKE (*Reynoutria japonica;* Bangor University) (4.5%); iii) T66 - Yeast-based formulation (1%; BioAtlantis Ltd.); iv) T90 - Yeast-based formulation (1%; BioAtlantis Ltd.); v) conventional treatment - CT (4.8% (p/p) cymoxanil + 40% (p/p) folpet + 8% (p/p) metalaxyl (SAPEC) or 10.1% (p/p) penconazole (SAPEC) or 50% (p/p) tebuconazole + 25% (p/p) trifloxystrobin (BAYER) or 8% (p/p) cymoxanil + 66% (p/p) folpet (ASCENZA) or 50% (p/p) kresoxim-methyl (BASF)) (prepared according to the manufacturer guidelines), and vi) control - C (water) (**Supplementary Tables 1, 2**). T66 and T90 are proprietary biological formulations derived from microbial fermentation, developed by BioAtlantis Ltd. These formulations contain proprietary strains of yeast: in formulation T66 exhibits moderate to low tolerance to salt, whereas the strain in formulation T90 demonstrates a high tolerance, capable of withstanding sodium chloride concentrations of up to 25%. The strains were suspended in water along with a stabilizing agent.

The applications were made in 2020 (NE20, JKE20, T6620, T9020, CT20, C20) (**Supplementary Table 1**) and in 2021 (NE21, JKE21, T6621, T9021, CT21, C21) (**Supplementary Table 2**). At harvest (BBCH 89), leaves were sampled directly to liquid nitrogen, taken from the three randomized blocks of six plants in each of the six treatment conditions. The samples were stored at –80°C until analysis.

### 2.2 DNA extraction

DNA was extracted from leaves using the DNeasy^®^ Plant Mini Kit (Qiagen, Hilden, Germany), according to the manufacturer’s protocol. Briefly, leaf material was macerated in liquid nitrogen, and about 100 mg of leaf powder was used. DNA concentration was determined by UV spectrometer (Nanodrop^®^ ND-1000, Thermo Fisher Scientific, USA) and stored at -20°C until metagenomic analysis.

### 2.3 Metagenomic analysis

For microbial community profiling, DNA samples were sent to Novogene Co., Ltd. (Cambridge, UK) for high-throughput sequencing. The bacterial and fungal communities were characterized through 16S rRNA gene (V3-V4), ITS1 and ITS2 regions sequencing, respectively (**Supplementary Table 3**). Library preparation, sequencing, and initial quality control were performed by Novogene according to standard protocols. The sequencing was conducted on the Illumina platform, using 2 × 250 bp paired-end chemistry to generate raw reads. The resulting raw data were processed using QIIME1 for quality filtering, OTU clustering and taxonomic classification.

### 2.4 Data analysis

Diversity metrics, including observed species richness and the Shannon-Weiner diversity index, were calculated using PAST v4.03 (Hammer et al., 2001) to assess within-sample microbial diversity. The Krona charts were performed according to the method described by Ondov et al. (2013) for visualizing and exploring the taxonomic composition of metagenomic data. Differences in community composition among sample groups were evaluated using PERMANOVA (Permutational Multivariate Analysis of Variance), also performed in PAST, based on Bray–Curtis dissimilarity matrices (Anderson, 2001). Further multivariate analyses were carried out using the Community Analysis Package v3.01 (CAP) (Clarke, 1993). Non-metric multidimensional scaling (NMDS) was used to visualize patterns in microbial community composition across samples. The Kruskal’s stress value was calculated to assess the goodness-of-fit of the NMDS ordination, with lower values indicating a better representation of the original dissimilarity data in reduced dimensions (Kruskal, 1964). Similarity Percentage (SIMPER) analysis was applied to identify the taxa contributing most to the observed differences between groups. Analysis of Similarities (ANOSIM) was used to statistically test the dissimilarity in community structure among predefined groups. ANOSIM generates a *p*-value (with significance considered at *p* < 0.05) and an R^2^, which quantify the degree of separation between groups: values close to 0 indicate little to no separation, while values approaching 1 suggest complete dissimilarity (Clarke, 1993). All statistical analyses were performed with a significant level of *p* < 0.05.

To explore relationships between bacterial and fungal taxa, a Spearman correlation analysis was performed between the relative abundances of bacterial and fungal genera across all treatments. Only genera representing more than 0.5% of the total relative abundance in the complete dataset were included. All statistical analyses were performed with a significant level of *p* < 0.05.

## 3 Results and discussion

### 3.1 Diversity and composition of fungal community

**Figure 1:**
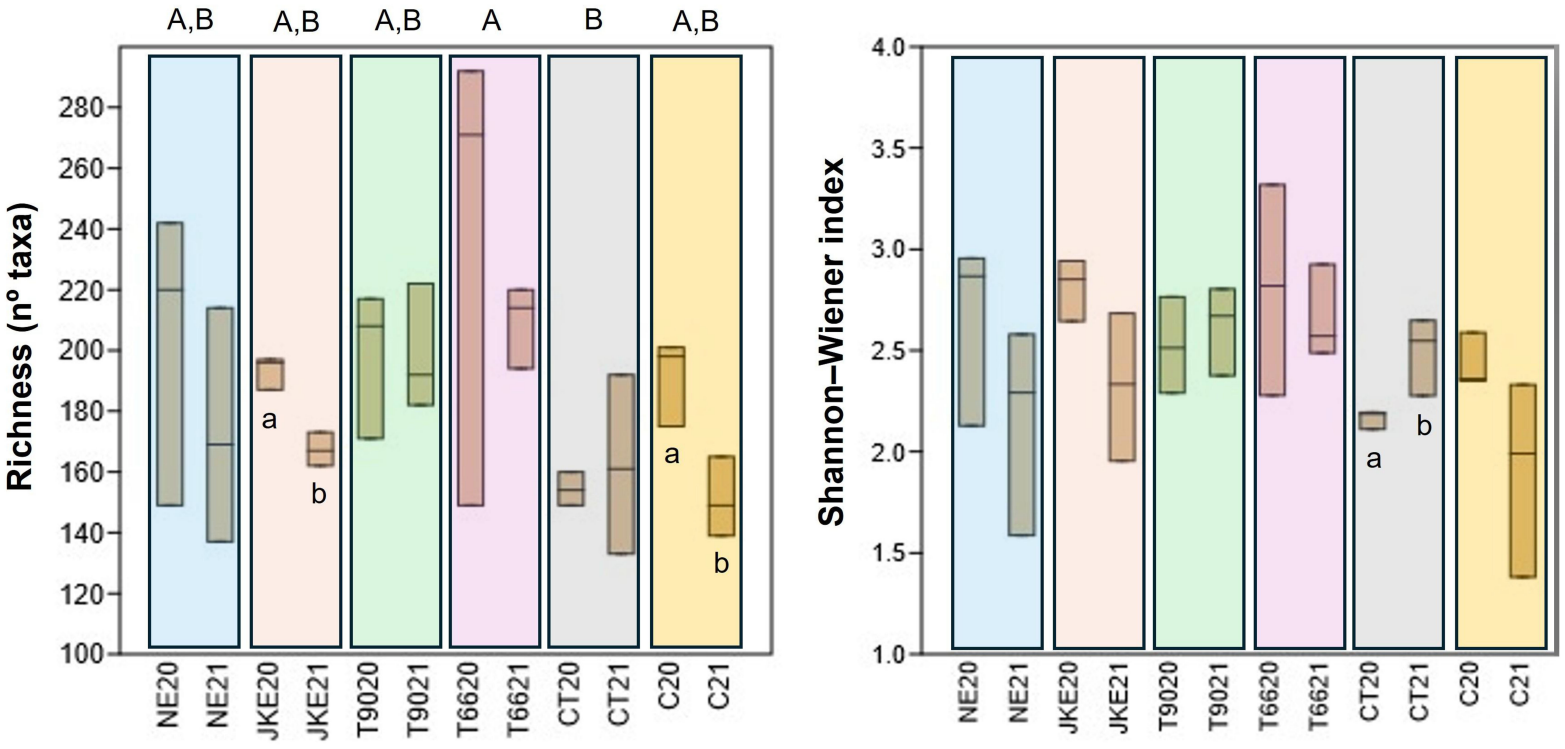
Boxplots showing the fungal community diversity (Richness and Shannon-Weiner index) of cv. ‘Touriga Franca’ under six different foliar treatments at harvest of 2020 and 2021. Different letters mean significant differences between treatments (uppercase) and differences between years for the same treatment (lowercase); no letters mean no-significant differences. NE, nettle extract; JKE, Japanese knotweed extract; T66, yeast extract; T99, yeast extract; CT, conventional treatment; C, control.

The richness (number of identified taxa) and the Shannon-Weiner index (**Figure 1**) were calculated to estimate fungal diversity. The treatments NE, T90, and T66 presented higher richness in both years compared to C suggesting that these treatments increased fungal diversity. In contrast, JKE20, JKE21, and CT20 have shown lower richness values indicating a reduction in taxa diversity under these conditions. Among all treatments, T6620 showed the highest richness, whereas CT20 consistently displayed the lowest, with the differences between these two treatments being statistically significant. Significant differences between years for richness were also observed in the treatments JKE and C.

No significant differences between treatments were detected for the Shannon-Weiner index. However, in terms of inter-annual variation in CT plants there was a significant increase in diversity from 2020 to 2021. The treatments CT and C presented lower Shannon-Weiner values compared to the other treatments, suggesting that plant- and yeast-based extracts contributed to enhance diversity of community balance.

**Figure 2:**
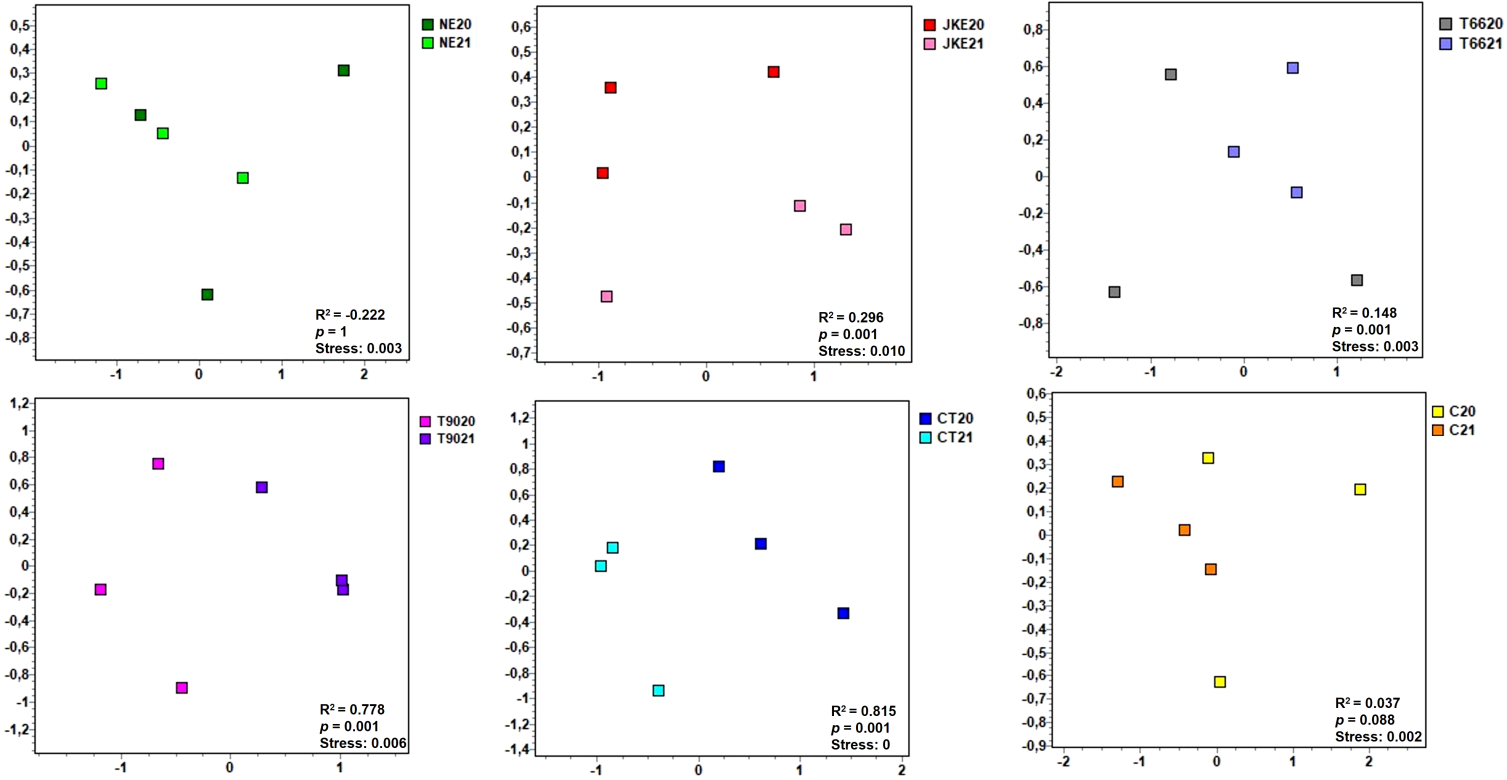
Non-metric multidimensional scaling (NMDS) plots of foliar fungal communities detected in cv. ‘Touriga Franca’ with six different foliar treatments at harvest of 2020 and 2021. Clustering analysis was performed with Bray-Curtis distance. Analysis of similarity (ANOSIM), based on Bray-Curtis distance and Kruskal’s stress values are displayed. NE, Nettle extract; JKE, Japanese knotweed extract; T66 and T90, Yeast extract; CT, Conventional treatment; C, Control.

To further investigate treatment and year effects on fungal community composition, Bray–Curtis dissimilarity analysis was applied and visualized via non-metric multidimensional scaling (NMDS) plots (**Figure 2**; **Supplementary Figure 2**), with PERMANOVA results shown in **Supplementary Table 4**. The PERMANOVA analysis revealed a significant effect of treatment (*p* = 0.0002), followed by year (*p* = 0.0015), explaining approximately 56% and 10% of the variation in fungal community composition, respectively (**Supplementary Table 4**). The interaction (treatment and year) had no influence in the fungal community composition (*p* = 0.998) (**Supplementary Table 4**). These findings were corroborated by NMDS plots, which clustered the samples according to treatment and year (**Figure 2**; **Supplementary Figure 2**).

In 2020 ANOSIM analysis indicated significant differences in fungal composition between treatments (R^2^ = 0.163, *p* = 0.004; **Supplementary Figure 2A**). The CT treatment differed significantly from all the others, while no significant differences were detected between the remaining treatments (**Supplementary Table 5**). In the SIMPER analysis, *Erysiphe necator* the causal agent of powdery mildew and one of the most damaging fungal diseases in grapevine (Wei et al., 2018; Gobbi et al., 2020), was identified as the species that most contributed to the differentiation between CT and treatments NE, T90, and C. For JKE and T66, *Aureobasidium pullulans* was the key species differentiating them from CT (**Supplementary Table 6**). *A. pullulans* has shown potential as a biocontrol agent against *Botrytis cinerea* the causal agent of bunch rot in grapevine (Karácsony et al., 2023).

In 2021, the differences between treatments were more pronounced (ANOSIM R^2^ = 0.418, *p* = 0.001; **Supplementary Figure 2B**). According to ANOSIM analysis treatments CT and C differed significantly from each other and from the other treatments, which revealed no significant difference among themselves (**Supplementary Table 7**). SIMPER analysis indicated as in 2020 that *E*. *necator* differentiated C from all the other treatments (JKE, T66, T90, CT) and also contributed to the differentiation of CT from NE, while *Sporobolomyces roseus* differentiated CT from T66 and T90 (**Supplementary Table 8**). *S*. *roseus* has been reported to reduce post-harvest diseases in apples (Janisiewicz et al., 1994), suggesting potential applications in grapevine protection.

When comparing the inter-annual variation for each treatment NE (ANOSIM R^2^ = -0.222, *p* = 1) and C (ANOSIM R^2^ = -0.037, *p* = 0.088) showed no significant differences in fungal composition. T66 (ANOSIM R^2^ = 0.148, *p* = 0.001) displayed minimal differences (**Figure 2**). JKE (ANOSIM R^2^ = 0.296, p = 0.001) showed moderate inter-annual variation, while T90 (ANOSIM R^2^ = 0.778, *p* = 0.001) and CT (ANOSIM R^2^ = 0.815, *p* = 0.001) exhibited the highest variation (**Figure 2**). SIMPER analysis indicated that *E*. *necator* was the species that differentiated the years in JKE, T66, T90 and C, whereas in CT, the key differentiating species was *A*. *pullulans* (**Supplementary Table 9**).

**Figure 3:**
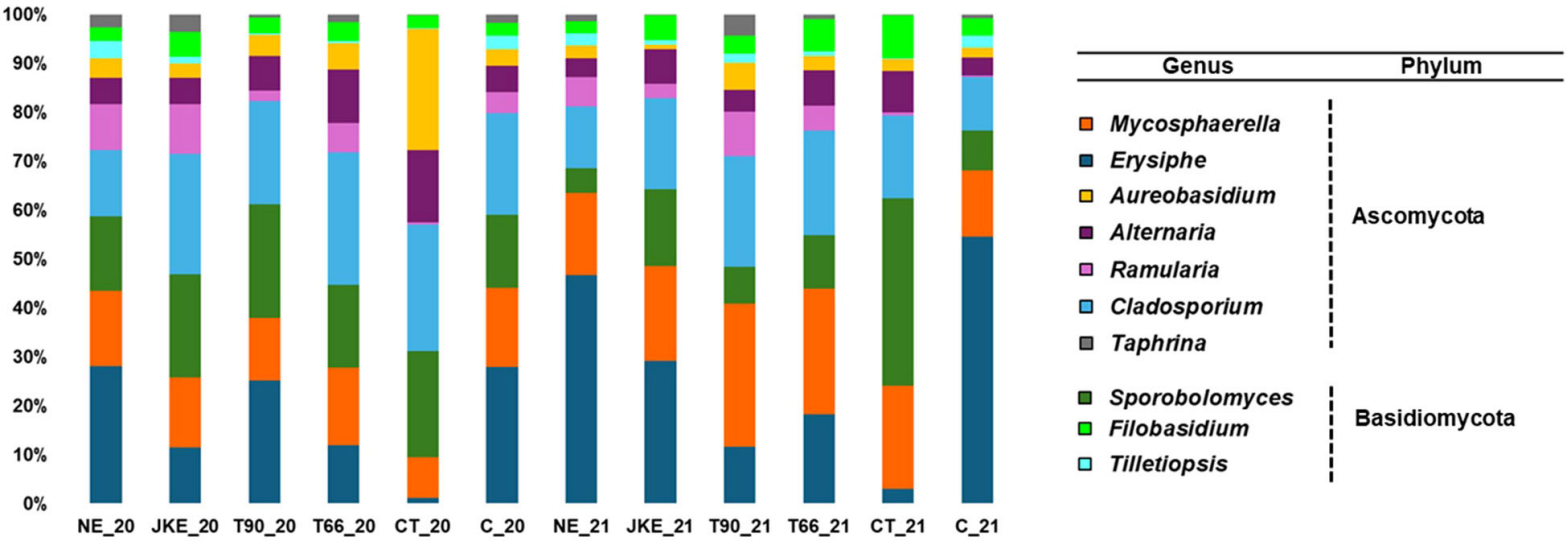
Relative abundance of fungal community (at genus level) detected in cv. ‘Touriga Franca’ with six different foliar treatments, at harvest of 2020 and 2021. Only the top 10 fungal genera are presented. NE, Nettle extract; JKE, Japanese knotweed extract; T66 and T90, Yeast extract; CT, Conventional treatment; C, Control.

A total of five fungal phyla were identified in both years (**Supplementary Figure 3**). The phylum Chytridiomycota was exclusive in 2020 and the Glomeromycota exclusive in 2021. Both years have four phyla in common. Ascomycota and Basidiomycota were the predominant phyla in all treatments in both years (**Supplementary Figure 3**), aligning with previous studies on grapevine leaves fungal diversity (Zhang et al., 2017; Miliordos et al., 2021). A total of 100 fungal families were found in 2020 and 98 in 2021 (**Supplementary Figure 4**). At the family level, treatments CT20, CT21, and C21 were distinct from the others, showing a different taxonomic composition compared to the remaining treatments (**Supplementary Figure 4**). Marked inter-annual variation in fungal family abundance was observed, particularly in NE, T90, CT and C (**Supplementary Figure 4**). At the genus level 90 genera were identified in 2020 and 71 in 2021 (**Figure 3**), with the top ten genera including *Erysiphe*, *Cladosporium*, *Mycosphaerella* and *Sporobolomyces* (**Figure 3**; **Supplementary Figure 5**) in both years. *Erysiphe* was notably dominant, containing important phytopathogens such as *E*. *necator*, extremely important in viticulture as previously mentioned. The treatments NE, T90 and C, in 2020, and NE, JKE and C, in 2021, exhibited the highest relative abundance of *Erysiphe* (**Figure 3**). It was also verified that this genus was more predominant in 2021 than in 2020, which may have been influenced by meteorological, since 2021 was rainier than 2020 (**Supplementary Figure 1**) and powdery mildew infections are promoted by humidity (Belhadj et al., 2006). According to ADVID (2021) the mild temperatures recorded in the spring/summer period of 2021 created good conditions for the development of powdery mildew, that remained active, requiring in some situations a curative protection strategy until the end of the cycle.

### 3.2 Diversity and composition of bacterial community

**Figure 4:**
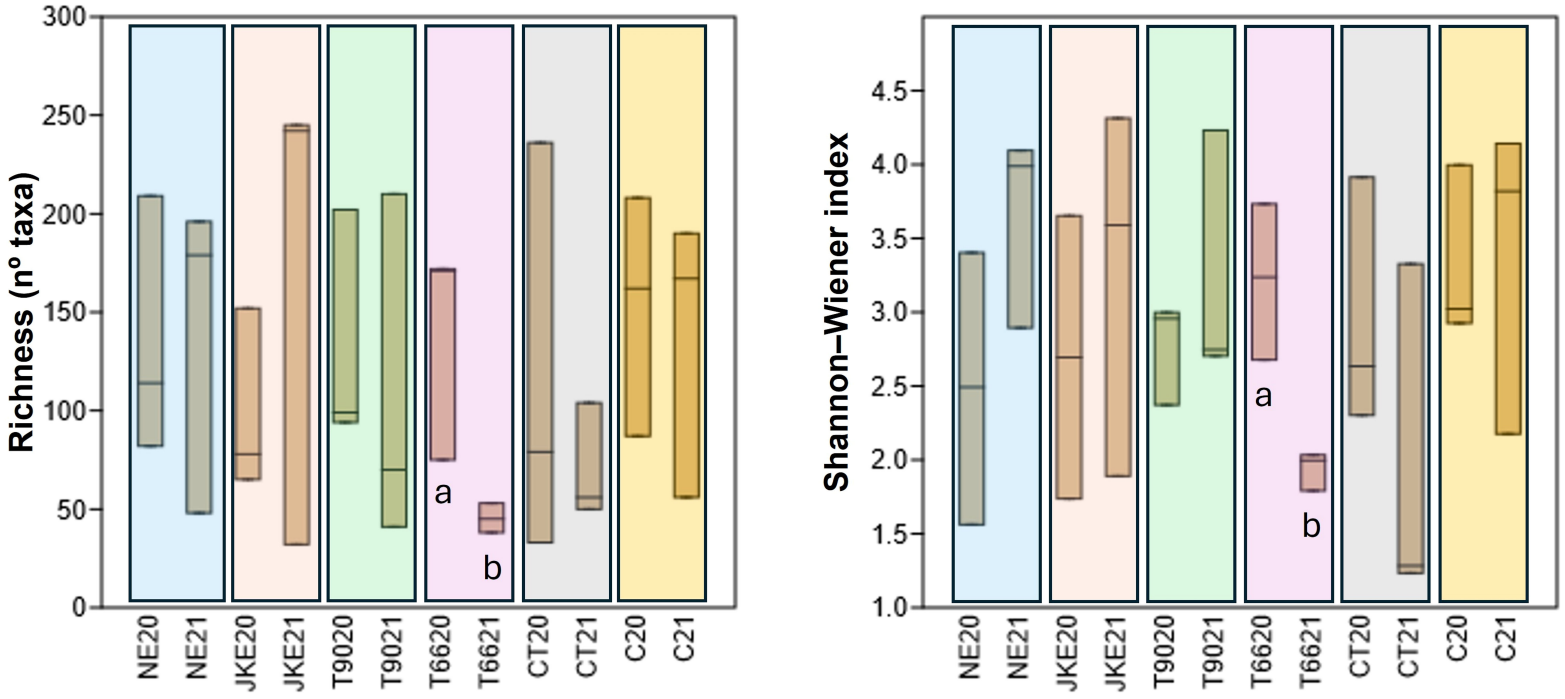
Boxplots showing the bacterial community diversity (Richness and Shannon-Weiner index) of cv. ‘Touriga Franca’ under six different foliar treatments at harvest of 2020 and 2021. Different letters mean significant differences between years for the same treatment; no letters mean no-significant differences. NE, Nettle extract; JKE, Japanese knotweed extract; T66 and T90, Yeast extract; CT, Conventional treatment; C, Control.

To assess the influence of plant- and yeast- based extracts on bacterial diversity, richness (number of identified taxa) and Shannon-Weiner index were calculated (**Figure 4**). In 2020 richness values were generally lower across treatments, with CT and C showing the highest values. In 2021 richness was higher in NE, JKE, and T90, and lower in T66, CT, and C. Treatments T66 showed a significant decrease in richness from 2020 to 2021.

Regarding Shannon-Weiner index (**Figure 4**) T66 also showed a significant decrease in diversity from 2020 to 2021, consistent with the richness results. In contrast, the remaining treatments (except CT) showed an opposite trend, with increases diversity in 2021, suggesting a possible effect of the year on the bacterial community structure.

PERMANOVA analysis based on Bray–Curtis dissimilarity revealed that treatment significantly influenced bacterial composition (*p* = 0.044), explaining about 52% of the variation (**Supplementary Table 10**). However, neither year (*p* = 0.080) nor the interaction between treatment and year (*p* = 0.920) significantly affected bacterial community composition (**Supplementary Table 10**).

The NMDS plot based on the Bray–Curtis (**Figure 4**; **Supplementary Figure 6**) showed that in 2020 there were no significant differences in bacterial composition between treatments (ANOSIM R^2^ = - 0.039, *p* = 0.791; **Supplementary Figure 6A**), as confirmed by ANOSIM (**Supplementary Table 11**). In 2021 significant differences between treatments were observed (ANOSIM R^2^ = 0.163, *p* = 0.004), with CT being the most dissimilar (**Supplementary Figure 6B**). ANOSIM results confirmed that CT differed significantly from the other treatments (NE, JKE, T66, T90 and C), while T66 was also different from NE, CT and C (**Supplementary Table 12**). SIMPER analysis indicated that the genus *Pseudomonas* contributed the most to the dissimilarity between CT and the other treatments (NE, T66, T90, C) (**Supplementary Table 13**). *Pseudomonas* spp. have demonstrated biocontrol potential against grapevine trunk diseases (Niem et al., 2020), downy mildew control (Sun et al., 2024), and *Botrytis cinerea*, by inducing plant defense mechanisms (Verhagen et al., 2010). For T66 the genus *Lactococcus* was responsible for its dissimilarity from treatments NE and C (**Supplementary Table 13**).

**Figure 5:**
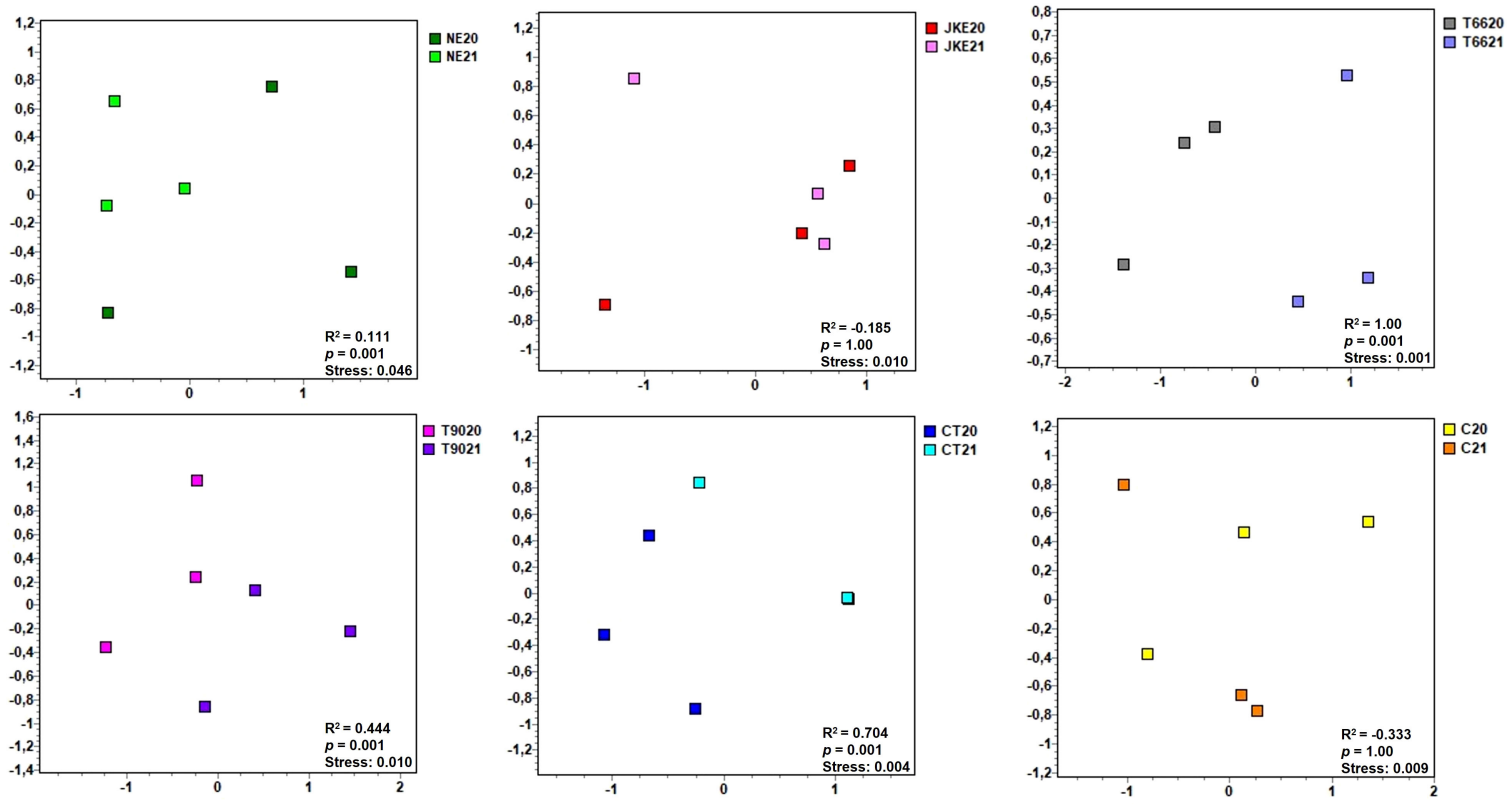
Non-metric multidimensional scaling (NMDS) plots of foliar bacterial communities detected in cv. ‘Touriga Franca’ with six different foliar treatments at harvest of 2020 and 2021. Clustering analysis was performed with Bray-Curtis distance. Analysis of similarity (ANOSIM), based on Bray-Curtis distance and Kruskal’s stress values are displayed. NE, Nettle extract; JKE, Japanese knotweed extract; T66 and T90, Yeast extract; CT, Conventional treatment; C, Control.

When comparing each treatment individually between years (**Figure 5**) no significant differences were found for JKE (ANOSIM R^2^ = -0.185, *p* = 1) and C (ANOSIM R^2^ = -0.333, *p* = 1). The NE treatment presented minor but statistically significant differences between years (ANOSIM R^2^ = 0.111, *p* = 0.001) (**Figure 5**). Moderate differences were observed for T90 (ANOSIM R^2^ = 0.444, *p* = 0.001) and CT (ANOSIM R^2^ = 0.704, *p* = 0.001) (**Figure 5**). The treatment T66 exhibited pronounced differences between years (ANOSIM R^2^ = 1.00, *p* = 0.001) (**Figure 5**). According to the SIMPER analysis (**Supplementary Table 14**) the main genera contributing to the differences between years were *Escherichia-Shigella* (NE), *Sphingomonas* (T66), *Delftia* (T90) and, *Pseudomonas* (CT). Notably, *Sphingomonas* and *Pseudomonas* are associated with plant health and productivity (Gobbi et al., 2020) and have been identified as consistent components of the grapevine microbiome, including in healthy, diseased, and recovered vines (Bulgari et al., 2014).

**Figure 6:**
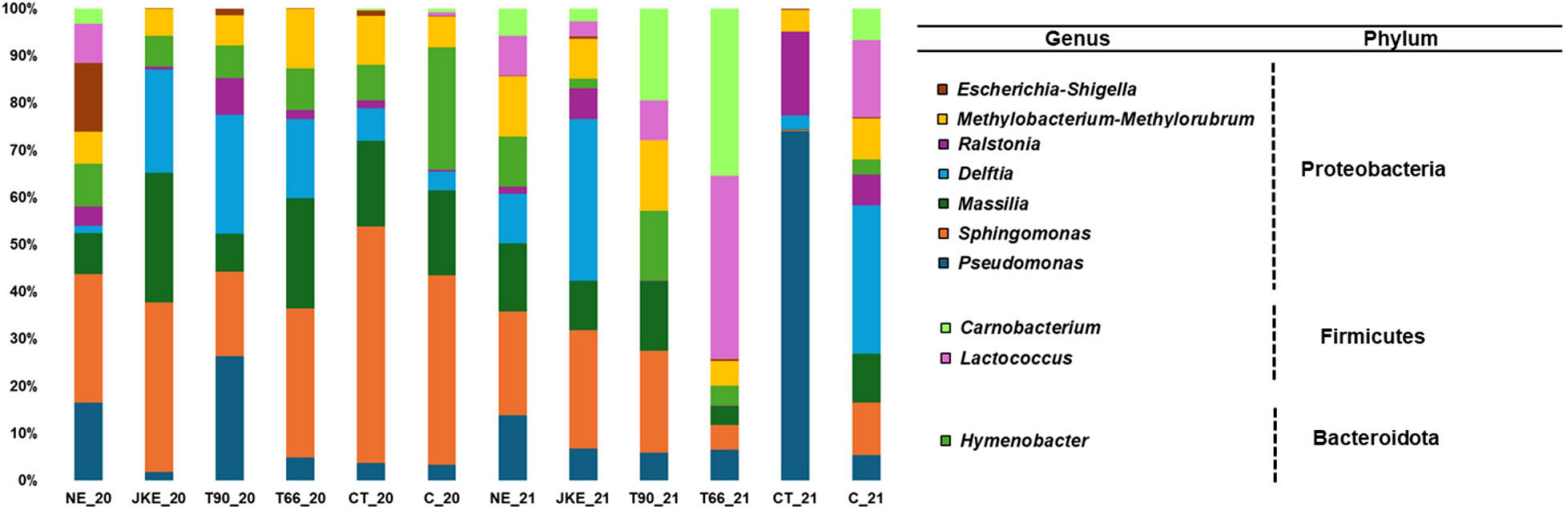
Relative abundance of bacterial community (at genus level) detected in cv. ‘Touriga Franca’ with different foliar treatments, at harvest of 2020 and 2021. Only the top 10 fungal genera are presented. NE, Nettle extract; JKE, Japanese knotweed extract; T66 and T90, Yeast extract; CT, Conventional treatment; C, Control.

A total of 14 bacterial phyla were detected in 2020 and 13 in 2021, 12 in common in both years (**Supplementary Figure 7**). The phyla Chloroflexie and Latescibacterota were exclusive in 2020 and Methylomirabilota in 2021. In both years, Proteobacteria and Firmicutes were the dominant phyla (**Supplementary Figure 7**), which is consistent with previous studies on grapevine leaves from different regions and cultivars (Leveau and Tech, 2011; ZarraonaIndia et al., 2015; Wei et al., 2018; Gobbi et al., 2020). At family level 76 were found in 2020 and 73 in 2021, with 60 shared between both years (**Supplementary Figure 8**). At the genus level 164 bacteria genera were detected in 2020 and 160 in 2021, with 128 shared between the two years (**Figure 6**). Overall, the most abundant genera were *Pseudomonas*, followed by *Sphingomonas* (**Figure 6**; **Supplementary Figure 9A**), as previously reported in grapevine leaf microbiomes studies conducted in the field across different regions (California, Long Island and Xinjiang province) and cultivars (‘Chardonnay’, ‘Merlot’ and ‘Cabernet Sauvignon’) (Leveau and Tech, 2011; ZarraonaIndia et al., 2015; Wei et al., 2018), as well as *in vitro* studies (Cappelletti et al., 2016). According to the Krona charts, *Sphingomonas* was the most abundant genus in 2020, whereas in 2021 was *Pseudomonas* (**Supplementary Figures 9B, C**). NE was the treatment with the highest number of detected bacterial genera in 2020, while JKE presented the highest genera richness in 2021 (**Figure 6**).

The mechanisms by which plant- and yeast-based extracts modulate the grapevines natural defenses, as well as their general ecological impacts, particularly on the grapevine leaf microbiome, remain insufficiently explored. Therefore, in this study, we investigated the effect of biofungicides derived from plant (NE and JKE) and yeast (T66 and T90) extracts on the grapevine leaf microbiome. In this study it was possible to observe that both ‘treatment’ and ‘year’ influenced the composition of the fungal and bacterial leaves communities. Regarding the fungal community the differences can be seen in the Krona charts (**Supplementary Figure 5**) and in the relative abundance graphs (**Figure 3**; **Supplementary Figures 3, 4**). Among the treatments, CT appears to have the greatest influence showing the lowest richness and diversity compared to the treatments under study (NE, JKE, T66, T90) and also with C (**Figure 1**). Regarding the effect of the year, a larger variation was observed particularly in the NE, T90, CT, and C treatments, as evidenced by significant changes in family (**Supplementary Figure 4**) and genus abundance (**Figure 3**) between 2020 and 2021. A higher richness was observed in the bacteria community compared to the fungal community, consistent with the findings of Zhang et al. (2017) in a study on grapevine leaves (cv. ‘Kyoho’) in Beijing. The CT21 treatment showed the strongest impact on bacterial community composition, confirmed by NMDS plots, which show its clear separation from the other treatments and also supported by the ANOSIM test (**Supplementary Figure 6B**; **Supplementary Table 12**). This impact may be explained by the fact that conventional treatment (CT) is based on synthetic chemicals, and that the frequency of application of broad-spectrum fungicides may affect not only pathogens, but also beneficial or neutral microorganisms present on grapevine leaves. In contrast, the plant- and yeast-based treatments used in this study are generally considered more favorable to microbial communities. These formulations contain bioactive molecules with antifungal activity, including elicitors that can activate plant defense mechanisms. Thus, instead of acting directly on the microbiome, their effects are mainly related to the plant’s immune responses. The T6621 treatment presented a difference in bacterial composition (**Figure 6**) especially in relation to NE21 and C21 treatments, and it is possible to observe in NMDS plots its difference in relation to these two treatments (**Supplementary Figure 6B**), which is also confirmed by the ANOSIM and SIMPER (**Supplementary Tables 11, 12**). The influence of ‘treatment’ and ‘year’ on the bacterial community was also evident in the Krona charts (**Supplementary Figure 9**) and in the relative abundance graphs (**Figure 6**; **Supplementary Figures 7, 8**). As previously mentioned, *Sphingomonas* was the most abundant genus in 2020 and *Pseudomonas* in 2021 (**Figure 6**), and both genera are known for their roles in plant health and productivity. These findings suggest that bacterial community is more sensitive to the inter-annual variation than the fungal community which appears to be more resilient and does not modify its composition over the years. The decision to perform sampling over two consecutive years aimed to capture the effect of interannual environmental variability on the grapevine microbiome. Previous studies have shown that microbial community composition, abundance, and distribution, can be influenced by climate conditions, geographical location, host plant phenology, physical and chemical properties, phyllosphere or soil characteristics, vineyard management practices, and the grapevine cultivar (Azevedo-Silva et al., 2021; Castanera et al., 2024). In our study, we observed substantial differences in microbial composition, particularly in the control treatment (C), across the two years. These differences, evident at various taxonomic levels (**Figures 3**, **6**; **Supplementary Figures 3, 4, 7, 8**), suggest that microbial communities may undergo reorganization in response to changing environmental conditions. Although the main focus of the study was not to track adaptation mechanisms, these results provide initial evidence that such adaptive ecological responses may occur. This aspect could be explored in more depth in future long-term studies.

Some studies also verified that alternative treatments to conventional fungicides can modulate the bacterial community on grapevine leaves without significantly altering the fungal community, which appears to be more resilient and mainly influenced by seasonality (Gobbi et al., 2020; Miliordos et al., 2021). These findings are consistent with the results of the present study, where bacterial communities responded more markedly to treatment and year variation, particularly under CT and T6621, while fungal communities showed greater stability across years. This reinforces the notion that eco-friendly treatments may shape the bacterial communities on the leaf surface without disrupting the natural fungal balance, a key factor when designing sustainable plant protection strategies.

In this study, Spearman correlation analysis between the relative abundances of bacterial and fungal genera was performed for each treatment (**Supplementary Figure 10**). In the NE treatment, a negative correlation was observed between the fungal genus *Stemphylium* and several bacterial genera (**Supplementary Figure 10A**). In the JKE treatment, the genus *Tillietiopsis* showed negative correlations with a significant number of bacterial genera (**Supplementary Figure 10B**). For the T66 treatment, *Symmetrospora* exhibited positive correlations with some bacteria genera, whereas *Filobasidium* showed negative correlations (**Supplementary Figure 10C**). In the case of the T90 treatment, the fungal genera *Alternaria, Mycosphaerella*, and *Erysiphe* showed predominantly positive correlations with multiple bacterial genera, while *Taphrina*, *Ramularia*, *Neosetophoma*, and *Cladosporium* showed mainly negative correlations with various bacterial genera (**Supplementary Figure 10D**). In contrast, only a few correlations were detected in the C and CT treatments, suggesting a more limited interaction between bacterial and fungal genera in these treatments (**Supplementary Figure 10E, F**).

## 4 Conclusion

Understanding microbial communities is crucial due to their role in plant protection and growth. The structure and dynamics of these communities are known to be influenced by environmental factors such as geography, grapevine cultivar, and climate. In this study, we demonstrated that alternative treatments to synthetic fungicides could modulate the microbial composition of grapevine leaves, particularly the bacterial community. These shifts may contribute to enhanced disease control and increased plant resilience, highlighting the potential of sustainable strategies in viticulture.

## Data Availability

The original contributions presented in the study are included in the article/**Supplementary Material**. Further inquiries can be directed to the corresponding author.
