# Supplementary material for: Metagenomic analysis of the effects of plant- and yeast-based formulations on the grapevine leaf microbiome of cv. ‘Touriga Franca’
Source: Front Plant Sci. 2025 Aug 14;16:1637143. doi: 10.3389/fpls.2025.1637143 (PMC12391083; doi:10.3389/fpls.2025.1637143)
Supplement: Supplementary file 1 [file DataSheet1.docx]

Supplementary Material

**
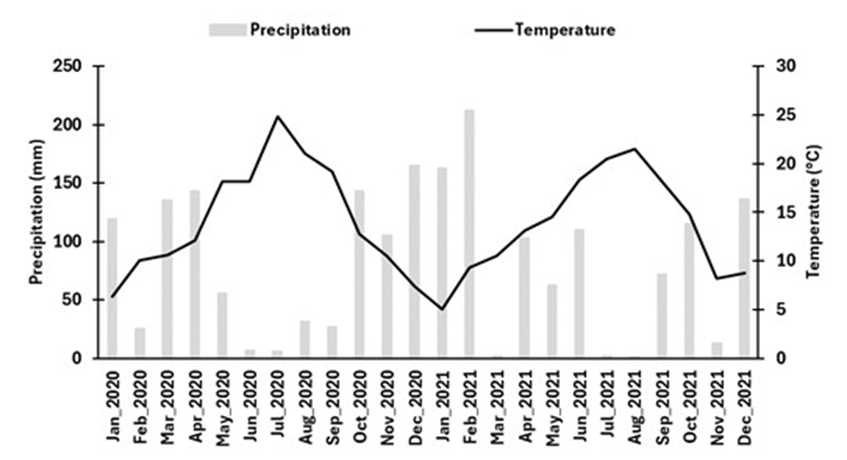
**

**Supplementary Figure 1.** Monthly average values of temperature and precipitation in the experimental vineyard, located at the University of Trás-os-Montes e Alto Douro, Baixo Corgo (Lower Corgo) sub-region of the Douro Demarcated Region, Vila Real, Northern Portugal, during 2020 and 2021.

**Supplementary Table 1.** Foliar application details, including the six treatments, dosages, and the growth phases during which the applications were conducted in 2020 growing season.

| **Phenological Stage** | **Treatmen** |
| --- | --- |
| Influorescence visible | Nettle extract (3%)  Japanese knotweed extract(4.5%)  T66 (Yeast-based formulation) (1%)  T90 (Yeast-based formulation) (1%)  Cymoxanil (4.8%) + folpet (40%) + metalaxyl (8%) (SAPEC)  Tebuconazole (50%) + trifloxystrobin (25%) (BAYER) |
| Inflorescence swelling | Nettle extract (3%)  Japanese knotweed extract(4.5%)  T66 (Yeast-based formulation) (1%)  T90 (Yeast-based formulation) (1%)  Penconazole (10.1%) (SAPEC)  Tebuconazole (50%) + trifloxystrobin (25%) (BAYER) |
| Inflorescences fully developed | Nettle extract (3%)  Japanese knotweed extract(4.5%)  T66 (Yeast-based formulation) (1%)  T90 (Yeast-based formulation) (1%)  Cymoxanil (4.8%) + folpet (40%) + metalaxyl (8%) (SAPEC)  Penconazole (10.1%) (SAPEC) |
| Flowers separating | Nettle extract (3%)  Japanese knotweed extract(4.5%)  T66 (Yeast-based formulation) (1%)  T90 (Yeast-based formulation) (1%)  Cymoxanil (4.8%) + folpet (40%) + metalaxyl (8%) (SAPEC)  Penconazole (10.1%) (SAPEC) |
| Flowering | Nettle extract (3%)  Japanese knotweed extract(4.5%)  T66 (Yeast-based formulation) (1%)  T90 (Yeast-based formulation) (1%)  Cymoxanil (4.8%) + folpet (40%) + metalaxyl (8%) (SAPEC)  Penconazole (10.1%) (SAPEC) |
| Full flowering: 50%  of flowerhoods fallen | Nettle extract (3%)  Japanese knotweed extract(4.5%)  T66 (Yeast-based formulation) (1%)  T90 (Yeast-based formulation) (1%)  Cymoxanil (4.8%) + folpet (40%) + metalaxyl (8%) (SAPEC)  Penconazole (10.1%) (SAPEC) |
| End of flowering | Nettle extract (3%)  Japanese knotweed extract(4.5%)  T66 (Yeast-based formulation) (1%)  T90 (Yeast-based formulation) (1%)  Cymoxanil (4.8%) + folpet (40%) + metalaxyl (8%) (SAPEC)  Penconazole (10.1%) (SAPEC) |
| Fruit set | Nettle extract (3%)  Japanese knotweed extract(4.5%)  T66 (Yeast-based formulation) (1%)  T90 (Yeast-based formulation) (1%) |
| Berries groat-sized | Nettle extract (3%)  Japanese knotweed extract(4.5%)  T66 (Yeast-based formulation) (1%)  T90 (Yeast-based formulation) (1%) |
| Berries pea-sized | Nettle extract (3%)  Japanese knotweed extract(4.5%)  T66 (Yeast-based formulation) (1%)  T90 (Yeast-based formulation) (1%) |
| Beginning of berry touch | Nettle extract (3%)  Japanese knotweed extract(4.5%)  T66 (Yeast-based formulation) (1%)  T90 (Yeast-based formulation) (1%) |
| Berry touch complete | Nettle extract (3%)  Japanese knotweed extract(4.5%)  T66 (Yeast-based formulation) (1%)  T90 (Yeast-based formulation) (1%) |
| Beginning of veraison | Nettle extract (3%)  Japanese knotweed extract(4.5%)  T66 (Yeast-based formulation) (1%)  T90 (Yeast-based formulation) (1%) |
| Veraison | Nettle extract (3%)  Japanese knotweed extract(4.5%)  T66 (Yeast-based formulation) (1%)  T90 (Yeast-based formulation) (1%) |

**Supplementary Table 2.** Foliar application details, including the six treatments, dosages, and the growth phases during which the applications were conducted in 2021 growing season.

| **Phenological Stage** | **Treatmen** |
| --- | --- |
| Leaves unfolded | Nettle extract (3%)  Japanese knotweed extract(4.5%)  T66 (Yeast-based formulation) (1%)  T90 (Yeast-based formulation) (1%) |
| Influorescence visible | Nettle extract (3%)  Japanese knotweed extract(4.5%)  T66 (Yeast-based formulation) (1%)  T90 (Yeast-based formulation) (1%) |
| Inflorescence swelling | Nettle extract (3%)  Japanese knotweed extract(4.5%)  T66 (Yeast-based formulation) (1%)  T90 (Yeast-based formulation) (1%) |
| Inflorescences fully developed | Nettle extract (3%)  Japanese knotweed extract(4.5%)  T66 (Yeast-based formulation) (1%)  T90 (Yeast-based formulation) (1%)  Cymoxanil (4.8%) + folpet (40%) + metalaxyl (8%) (SAPEC) |
| Flowers separating | Nettle extract (3%)  Japanese knotweed extract(4.5%)  T66 (Yeast-based formulation) (1%)  T90 (Yeast-based formulation) (1%)  Cymoxanil (4.8%) + folpet (40%) + metalaxyl (8%) (SAPEC |
| Flowering | Nettle extract (3%)  Japanese knotweed extract(4.5%)  T66 (Yeast-based formulation) (1%)  T90 (Yeast-based formulation) (1%)  Cimoxanil (8%) + folpet (66)% (ASCENZA)  Kresoxim-methyl (50%) (BASF) |
| Full flowering: 50%  of flowerhoods fallen | Nettle extract (3%)  Japanese knotweed extract(4.5%)  T66 (Yeast-based formulation) (1%)  T90 (Yeast-based formulation) (1%) |
| Fruit set | Nettle extract (3%)  Japanese knotweed extract(4.5%)  T66 (Yeast-based formulation) (1%)  T90 (Yeast-based formulation) (1%)  Cimoxanil (8%) + folpet (66)% (ASCENZA)  Kresoxim-methyl (50%) (BASF) |
| Berries pea-sized | Nettle extract (3%)  Japanese knotweed extract(4.5%)  T66 (Yeast-based formulation) (1%)  T90 (Yeast-based formulation) (1%) |
| Berry touch complete | Nettle extract (3%)  Japanese knotweed extract(4.5%)  T66 (Yeast-based formulation) (1%)  T90 (Yeast-based formulation) (1%)  Cimoxanil (8%) + folpet (66)% (ASCENZA)  Kresoxim-methyl (50%) (BASF) |
| Beginning of veraison | Nettle extract (3%)  Japanese knotweed extract(4.5%)  T66 (Yeast-based formulation) (1%)  T90 (Yeast-based formulation) (1%) |
| Veraison | Nettle extract (3%)  Japanese knotweed extract(4.5%)  T66 (Yeast-based formulation) (1%)  T90 (Yeast-based formulation) (1%)  Cimoxanil (8%) + folpet (66)% (ASCENZA)  Kresoxim-methyl (50%) (BASF) |

**Supplementary Table 3.** Primer sequences used for the characterization of bacterial and fungal communities.

| **Species Type** | **Amplified region** | **Primer** |
| --- | --- | --- |
| Bacteria | 16Sv4 | GTGCCAGCMGCCGCGGTAA,GGACTACHVGGGTWTCTAAT |
| Bacteria | 16Sv34 | CCTAYGGGRBGCASCAG,GGACTACNNGGGTATCTAAT |
| Bacteria | 16Sv45 | GTGCCAGCMGCCGCGGTAA,CCGTCAATTCCTTTGAGTTT |
| Bacteria | 16Sv57 | AACMGGATTAGATACCCKG,ACGTCATCCCCACCTTCC |
| Archaea | arcv45 | CAGCCGCCGCGGTAA,GTGCTCCCCCGCCAATTCCT |
| Archaea | arcv8 | TTWAGTCAGGCAACGAGC,TGTGCAAGGAGCAGGGAC |
| Eukaryote | 18Sv4 | GCGGTAATTCCAGCTCCAA,AATCCRAGAATTTCACCTCT |
| Eukaryote | 18Sv9 | CCCTGCCHTTTGTACACAC,CCTTCYGCAGGTTCACCTAC |
| Fungi | ITS1-1F | CTTGGTCATTTAGAGGAAGTAA,GCTGCGTTCTTCATCGATGC |
| Fungi | ITS1-5F | GGAAGTAAAAGTCGTAACAAGG,GCTGCGTTCTTCATCGATGC |
| Fungi | ITS2 | GCATCGATGAAGAACGCAGC,TCCTCCGCTTATTGATATGC |

**Fungi**

**Supplementary Table 4.** Pairwise comparisons PERMANOVA (R^2^ and *p*-value) of the fungal community composition between the six different treatments and year

|  | R^2^ | *p* |
| --- | --- | --- |
| **Treatment** | 0.5557 | 0.0002 |
| **Year** | 0.0993 | 0.0015 |
| **Treatment x Year (Interaction)** | -0.9880 | 0.9986 |


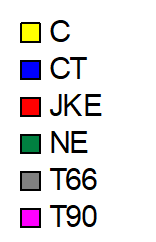

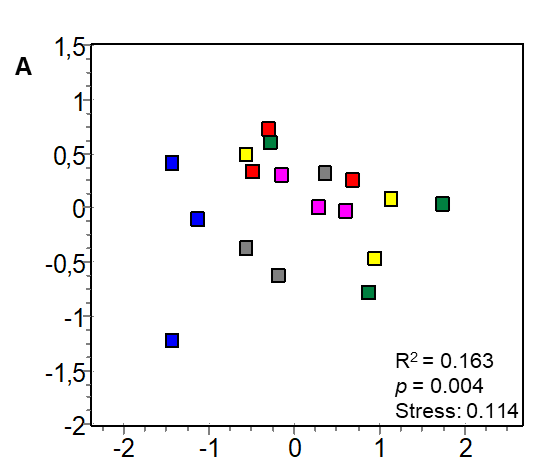

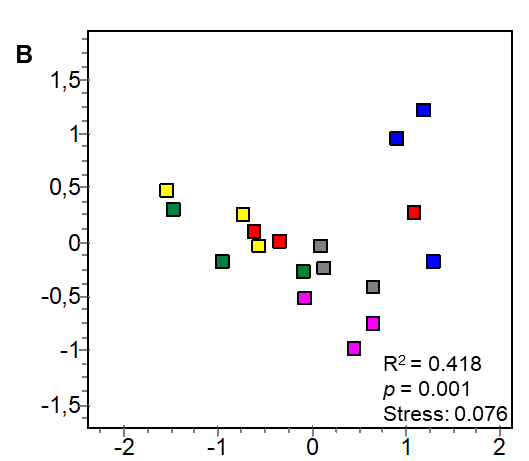


**Supplementary Figure 2.** Non-metric multidimensional scaling (NMDS) plots of foliar fungal communities detected in cv. ‘Touriga Franca’ with six different foliar treatments at harvest of 2020 (A) and 2021 (B). Clustering analysis was performed with Bray-Curtis distance. Analysis of similarity (ANOSIM), based on Bray-Curtis distance and Kruskal’s stress values are displayed. NE – Nettle extract; JKE – Japanese knotweed extract; T66 and T90 – Yeast extract; CT – Conventional treatment; C – Control.

**Supplementary Table 5.** Analysis of similarity (ANOSIM), based on Bray-Curtis distance, of the foliar fungal communities between six different treatments in the cv. ‘Touriga Franca’ at harvest 2020. Included are the R-statistics (R^2^) and p-values * (p ≤0.05); **( p ≤ 0.01); *** (p ≤ 0.001), no asterisk indicates p > 0.05. NE – Nettle extract; JKE – Japanese knotweed extract; T66 and T90 – Yeast extract; CT – Conventional treatment; C – Control.

|  | **C** | **CT** | **JKE** | **NE** | **T66** | **T90** |
| --- | --- | --- | --- | --- | --- | --- |
| **C** |  | 0.778* | -0.111 | -0.370 | -0.222 | -0.111 |
| **CT** | 0.778* |  | 0.741* | 0.704* | 0.556* | 0.815* |
| **JKE** | -0.111 | 0.741* |  | 0.037 | -0.185 | 0.111 |
| **NE** | -0.370 | 0.704* | 0.037 |  | -0.111 | 0.000 |
| **T66** | -0.222 | 0.556* | -0.185 | -0.111 |  | -0.037 |
| **T90** | -0.111 | 0.815* | 0.111 | 0.000 | -0.037 |  |

**Supplementary Table 6.** Foliar Similarity Percentage Analysis (SIMPER) of the foliar fungal community in 2020, based on Bray-Curtis distance, between six different treatments. The contributions of the most important species are presented, with a focus on treatments that showed statistical significance in ANOSIM. NE – Nettle extract; JKE – Japanese knotweed extract; T66 and T90 – Yeast extract; CT – Conventional treatment; C – Control.

| **Comparison** | **Cumulative %** | **Species** |
| --- | --- | --- |
| CT vs C | 22.34 | *Erysiphe necator* |
|  | 40.40 | *Aureobasidium pullulans* |
|  | 50.01 | *Sporobolomyces roseus* |
| CT vs JKE | 20.41 | *Aureobasidium pullulans* |
|  | 30.54 | *Alternaria alternata* |
|  | 39.81 | *Erysiphe necator* |
|  | 47.67 | *Cladosporium tenuissimum* |
| CT vs NE | 18.79 | *Erysiphe necator* |
|  | 34.61 | *Aureobasidium pullulans* |
|  | 45.78 | *Sporobolomyces roseus* |
| CT vs T66 | 20.29 | *Aureobasidium pullulans* |
|  | 30.51 | *Erysiphe necator* |
|  | 39.54 | *Cladosporium tenuissimum* |
|  | 48.14 | *Alternaria alternata* |
| CT vs T90 | 23.56 | *Erysiphe necator* |
|  | 43.83 | *Aureobasidium pullulans* |

**Supplementary Table 7.** Foliar Analysis of similarity (ANOSIM), based on Bray-Curtis distance, of the foliar fungal communities between six different treatments in the cv. ‘Touriga Franca’ at harvest 2021. Included are the R-statistics (R^2^) and p-values * (p ≤0.05); **( p ≤ 0.01); *** (p ≤ 0.001), no asterisk indicates p > 0.05. NE – Nettle extract; JKE – Japanese knotweed extract; T66 and T90 – Yeast extract; CT – Conventional treatment; C – Control.

|  | **C** | **CT** | **JKE** | **NE** | **T66** | **T90** |
| --- | --- | --- | --- | --- | --- | --- |
| **C** |  | 1.00* | 0.148* | -0.259 | 0.704* | 0.815* |
| **CT** | 1.00* |  | 0.481 | 0.926* | 0.777* | 0.926* |
| **JKE** | 0.148* | 0.481 |  | -0.148 | 0.185 | 0.296 |
| **NE** | -0.259 | 0.926* | -0.148 |  | 0.296 | 0.407 |
| **T66** | 0.704* | 0.777* | 0.185 | 0.296 |  | 0.111 |
| **T90** | 0.815* | 0.926* | 0.296 | 0.407 | 0.111 |  |

**Supplementary Table 8.** Similarity Percentage Analysis (SIMPER) of the foliar fungal community in 2021, based on Bray-Curtis distance, between six different treatments. The contributions of the most important species are presented, with a focus on treatments that showed statistical significance in ANOSIM. NE – Nettle extract; JKE – Japanese knotweed extract; T66 and T90 – Yeast extract; CT – Conventional treatment; C – Control.

| **Comparison** | **Cumulative %** | **Species** |
| --- | --- | --- |
| C vs CT | 42.73 | *Erysiphe necator* |
| C vs JKE | 39.02 | *Erysiphe necator* |
|  | 49.60 | *Cladosporium tenuissimum* |
| C vs T66 | 44.29 | *Erysiphe necator* |
| C vs T90 | 43.90 | *Erysiphe necator* |
| CT vs NE | 35.18 | *Erysiphe necator* |
| CT vs T66 | 24.15 | *Sporobolomyces roseus* |
|  | 41.02 | *Erysiphe necator* |
|  | 49.12 | *Basidiomycota* |
| CT vs T90 | 24.47 | *Sporobolomyces roseus* |
|  | 33.56 | *Erysiphe necator* |
|  | 41.52 | *Mycosphaerella tassiana* |
|  | 49.13 | *Basidiomycota* |

**Supplementary Table 9.** Similarity Percentage Analysis (SIMPER) of the different foliar fungal community in 2020 and 2021, based on Bray-Curtis distance, between six different treatments. The contributions of the most important species are presented, with a focus on treatments that showed statistical significance in ANOSIM. JKE – Japanese knotweed extract; T66 and T90 – Yeast extract; CT – Conventional treatment; C – Control.

| **Comparison** | **Cumulative %** | **Species** |
| --- | --- | --- |
| JKE20 vs JKE21 | 30.60 | *Erysiphe necator* |
|  | 39.71 | *Sporobolomyces roseus* |
|  | 48.18 | *Mycosphaerella tassiana* |
| T6620 vs T6621 | 21.60 | *Erysiphe necator* |
|  | 34.70 | *Mycosphaerella tassiana* |
|  | 44.62 | *Cladosporium tenuissimum* |
| T9020 vs T9021 | 19.50 | *Erysiphe necator* |
|  | 36.53 | *Mycosphaerella tassiana* |
| CT20 vs CT21 | 20.75 | *Aureobasidium pullulans* |
|  | 35.38 | *Sporobolomyces roseus* |
|  | 45.75 | *Cladosporium tenuissimum* |
| C20 vs C21 | 36.43 | *Erysiphe necator* |
|  | 48.18 | *Cladosporium tenuissimum* |


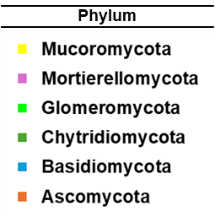

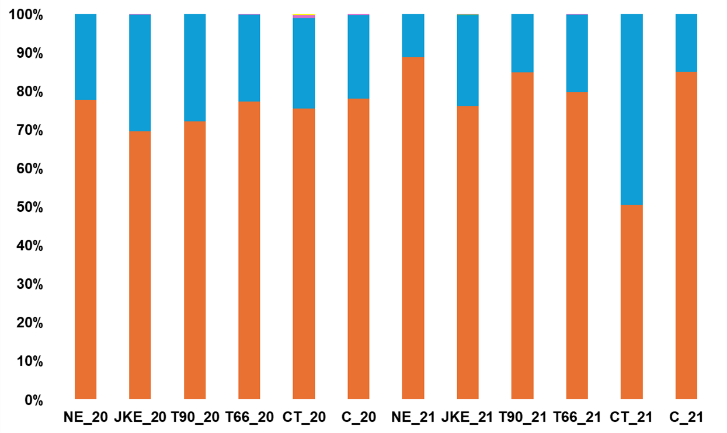


**Supplementary Figure 3.** Relative abundance of fungal community (at phylum level) detected in cv. ‘Touriga Franca’ with six different foliar treatments, at harvest of 2020 and 2021. NE – Nettle extract; JKE – Japanese knotweed extract; T66 and T90 – Yeast extract; CT – Conventional treatment; C – Control.


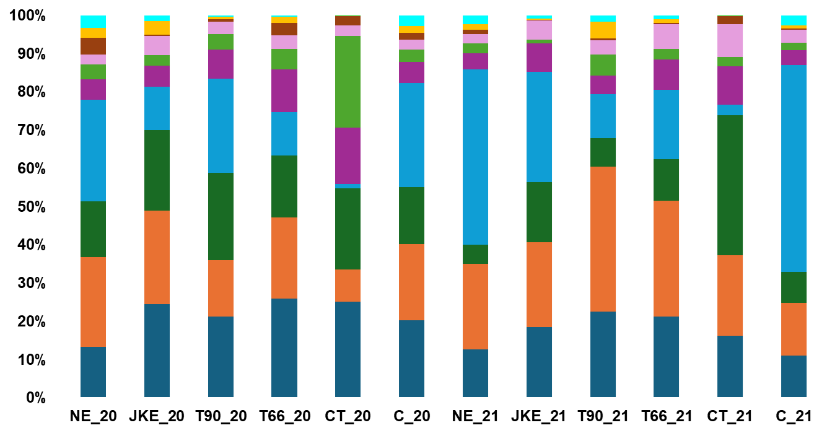

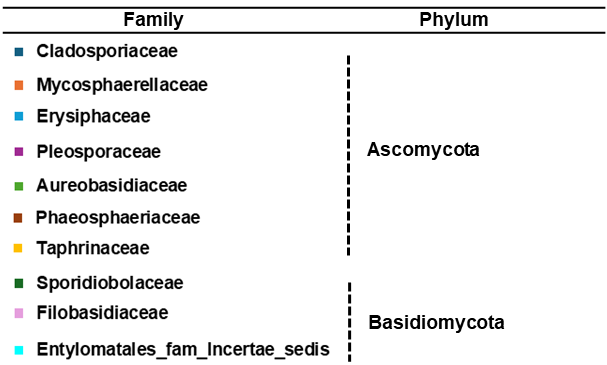


**Supplementary Figure 4.** Relative abundance of fungal community (at family level) detected in cv. ‘Touriga Franca’ with six different foliar treatments, at harvest of 2020 and 2021. Only the top 10 fungal families are present. NE – Nettle extract; JKE – Japanese knotweed extract; T66 and T90 – Yeast extract; CT – Conventional treatment; C – Control.


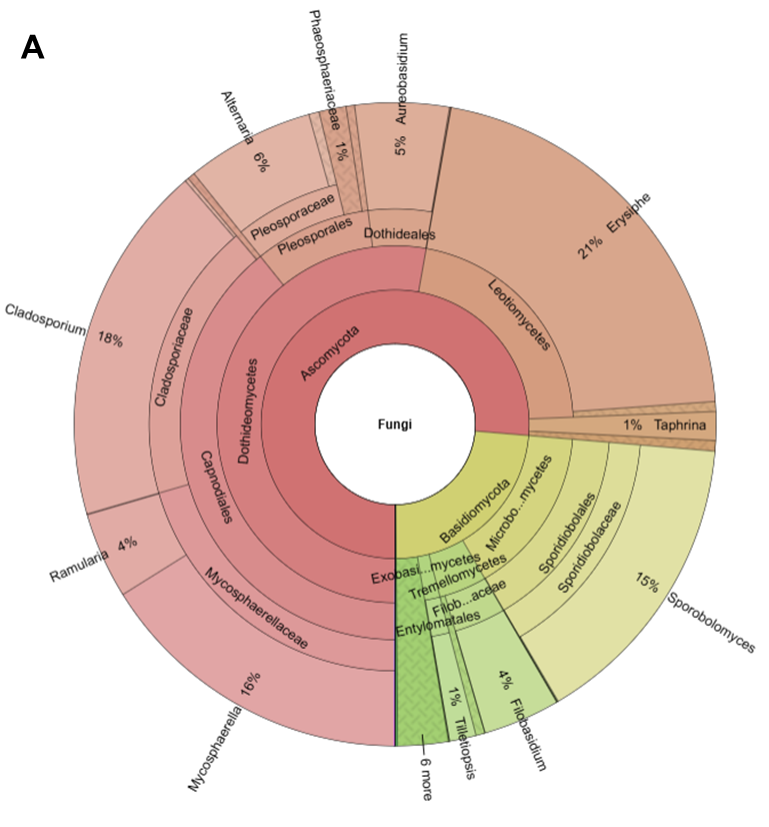


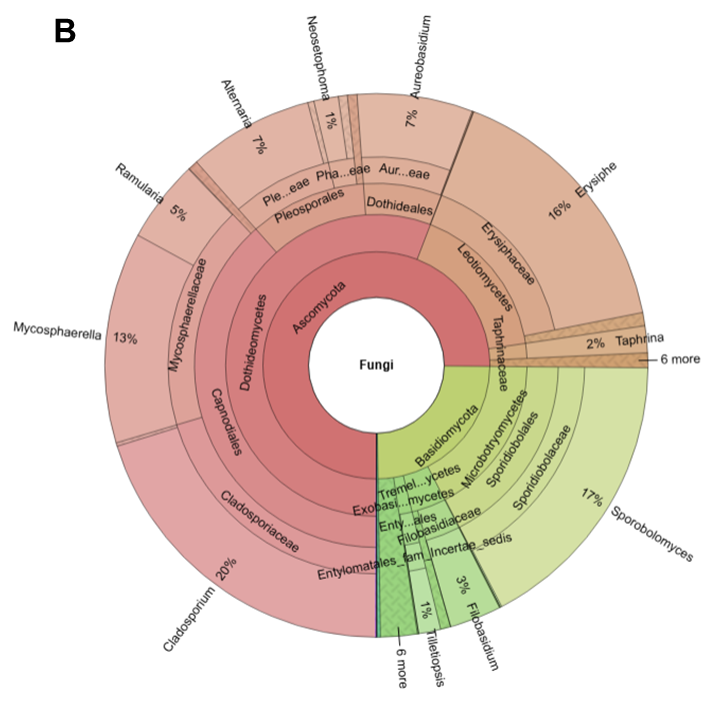


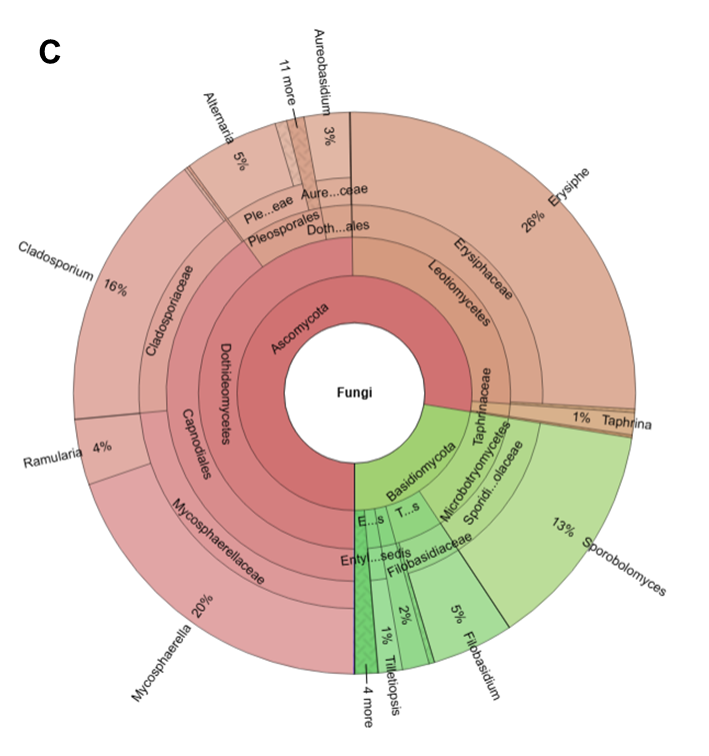


**Supplementary Figure 5.** Krona charts representing the relative abundance of the whole fungal communities detected in both years (A), in 2020 (B), and in 2021 (C) in all six treatments.

**Bacteria**

**Supplementary Table 10.** Pairwise comparisons PERMANOVA (R^2^ and *p*-value) of the bacterial community composition between the six different treatments and year.

|  | R^2^ | *p* |
| --- | --- | --- |
| **Treatment** | 0.522 | 0.044 |
| **Year** | 0.048 | 0.080 |
| **Treatment x Year (Interaction)** | -1.004 | 0.920 |


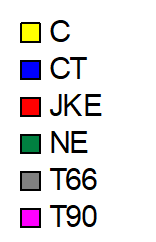

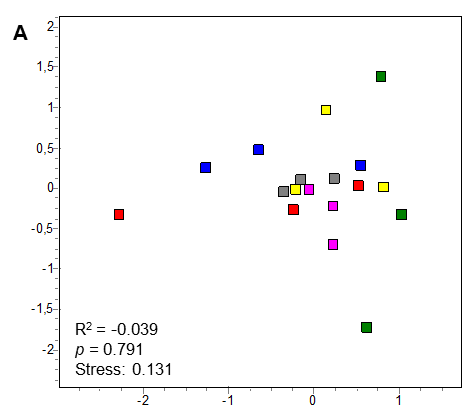

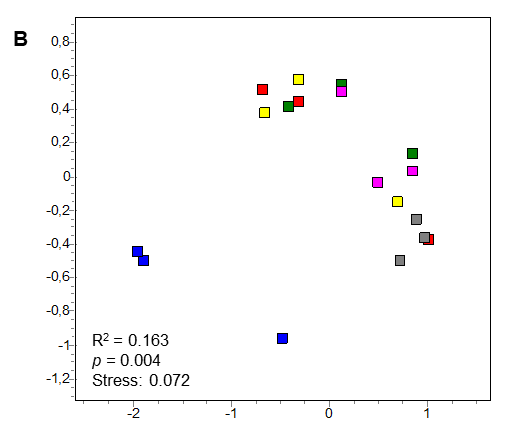


**Supplementary Figure 6.** Non-metric multidimensional scaling (NMDS) plots of foliar bacterial communities detected in cv. ‘Touriga Franca’ with six different foliar treatments at harvest of 2020 (A) and 2021 (B). Clustering analysis was performed with Bray-Curtis distance. Analysis of similarity (ANOSIM), based on Bray-Curtis distance and Kruskal’s stress values are displayed. NE – Nettle extract; JKE – Japanese knotweed extract; T66 and T90 – Yeast extract; CT – Conventional treatment; C – Control.

**Supplementary Table 11.** Analysis of similarity (ANOSIM), based on Bray-Curtis distance, of the foliar bacterial communities between six different treatments in the cv. ‘Touriga Franca’ at harvest 2020. Included are the R-statistics (R^2^) and p-values * (*p ≤*0.05); **( *p ≤* 0.01); *** (*p ≤* 0.001), no asterisk indicates p > 0.05. NE – Nettle extract; JKE – Japanese knotweed extract; T66 and T90 – Yeast extract; CT – Conventional treatment; C – Control.

|  | **C** | **CT** | **JKE** | **NE** | **T66** | **T90** |
| --- | --- | --- | --- | --- | --- | --- |
| **C** |  | -0.037 | -0.111 | -0.222 | -0.148 | -0.111 |
| **CT** | -0.037 |  | -0.074 | 0.037 | 0.148 | 0.074 |
| **JKE** | -0.111 | -0.074 |  | -0.111 | -0.074 | -0.148 |
| **NE** | -0.222 | 0.037 | -0.111 |  | 0.000 | -0.111 |
| **T66** | -0.148 | 0.148 | -0.074 | 0.000 |  | -0.074 |
| **T90** | -0.111 | 0.074 | -0.148 | -0.111 | -0.074 |  |

**Supplementary Table 12.** Analysis of similarity (ANOSIM), based on Bray-Curtis distance, of the foliar fungal communities between six different treatments in the cv. ‘Touriga Franca’ at harvest 2021. Included are the R-statistics (R^2^) and p-values * (p ≤0.05); **( p ≤ 0.01); *** (p ≤ 0.001), no asterisk indicates p > 0.05. NE – Nettle extract; JKE – Japanese knotweed extract; T66 and T90 – Yeast extract; CT – Conventional treatment; C – Control.

|  | **C** | **CT** | **JKE** | **NE** | **T66** | **T90** |
| --- | --- | --- | --- | --- | --- | --- |
| **C** |  | 0.593* | -0.333 | -0.407 | 0.519* | 0.000 |
| **CT** | 0.593* |  | 0.185 | 0.815* | 0.778* | 0.778* |
| **JKE** | -0.333 | 0.185 |  | -0.333 | 0.074 | 0.037 |
| **NE** | -0.407 | 0.815* | -0.333 |  | 0.519* | -0.037 |
| **T66** | 0.519* | 0.778* | 0.074 | 0.519* |  | 0.370 |
| **T90** | 0.000 | 0.778* | 0.037 | -0.037 | 0.370 |  |

**Supplementary Table 13.** Similarity Percentage Analysis (SIMPER) of the foliar bacterial community in 2021, based on Bray-Curtis distance, between six different treatments. The contributions of the most important genus are presented, with a focus on treatments that showed statistical significance in ANOSIM. NE – Nettle extract; T66 and T90 – Yeast extract; CT – Conventional treatment; C – Control.

| **Comparison** | **Cumulative %** | **Genus** |
| --- | --- | --- |
| CT vs C | 39.61 | *Pseudomonas* |
| CT vs NE | 38.88 | *Pseudomonas* |
| CT vs T66 | 41.42 | *Pseudomonas* |
| CT vs T90 | 39.03 | *Pseudomonas* |
| T66 vs C | 11.97 | *Lactococcus* |
|  | 21.40 | *Delftia* |
|  | 28.35 | *Carnobacterium* |
|  | 32.19 | *Latilactobacillus* |
|  | 35.82 | *Agathobacter* |
|  | 38.80 | *Collinsella* |
|  | 41.23 | *Methylobacterium-Methylorubrum* |
|  | 43.52 | *Sphingomonas* |
|  | 45.65 | *Catenibacterium* |
|  | 47.64 | *Massilia* |
|  | 49.52 | *Ralstonia* |
| T66 vs NE | 12.19 | *Lactococcus* |
|  | 21.81 | *Carnobacterium* |
|  | 27.20 | *Latilactobacillus* |
|  | 31.89 | *Akkermansia* |
|  | 35.92 | *Pseudomonas* |
|  | 39.74 | *Sphingomonas* |
|  | 42.99 | *Delftia* |
|  | 45.62 | *Collinsella* |
|  | 47.52 | *Serratia* |
|  | 49.30 | *Pseudomonas* |
|  | 50.97 | *Hymenobacter* |

**Supplementary Table 14.** Similarity Percentage Analysis (SIMPER) of the foliar bacterial community in 2020 and 2021, based on Bray-Curtis distance, between six different treatments. The contributions of the most important genus are presented, with a focus on treatments that showed statistical significance in ANOSIM. NE – Nettle extract; T66 and T90 – Yeast extract; CT – Conventional treatment; C – Control.

| **Comparison** | **Cumulative %** | **Genus** |
| --- | --- | --- |
| NE20 vs NE21 | 12.62 | *Escherichia-Shigella* |
|  | 25.19 | *Sphingomonas* |
|  | 35.51 | *Pseudomonas* |
|  | 45.09 | *Lactococcus* |
|  | 48.85 | *Carnobacterium* |
| T6620 vs T6621 | 15.27 | *Sphingomonas* |
|  | 26.69 | *Delftia* |
|  | 35.83 | *Lactococcus* |
|  | 44.37 | *Carnobacterium* |
| T9020 vs T9021 | 16.11 | *Delftia* |
|  | 28.24 | *Pseudomonas* |
|  | 36.22 | *Sphingomonas* |
|  | 41.29 | *Ralstonia* |
|  | 45.41 | *Carnobacterium* |
|  | 48.97 | *Latilactobacillus* |
| CT20 vs CT21 | 37.83 | *Pseudomonas* |


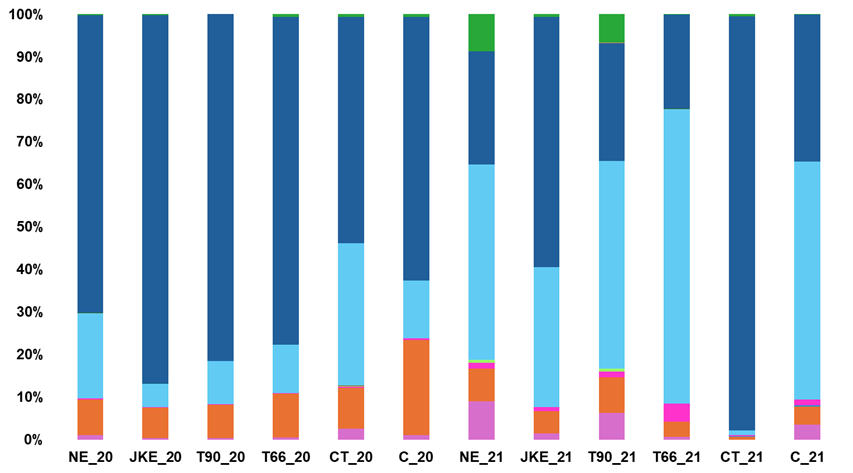

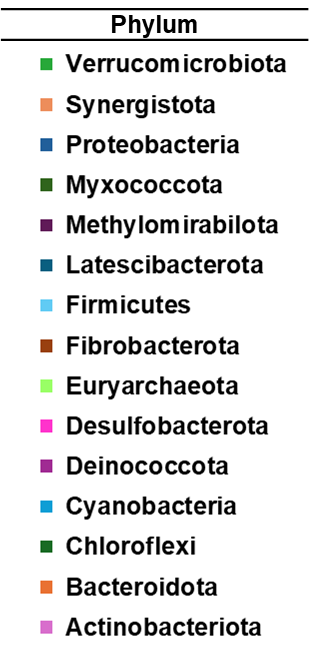


**Supplementary Figure 7.** Relative abundance of bacterial community (at phylum level) detected in cv. ‘Touriga Franca’ with six different foliar treatments, at harvest of 2020 and 2021. NE – Nettle extract; JKE – Japanese knotweed extract; T66 and T90 – Yeast extract; CT – Conventional treatment; C – Control.


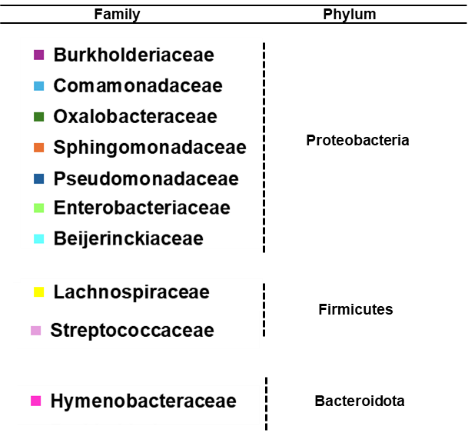

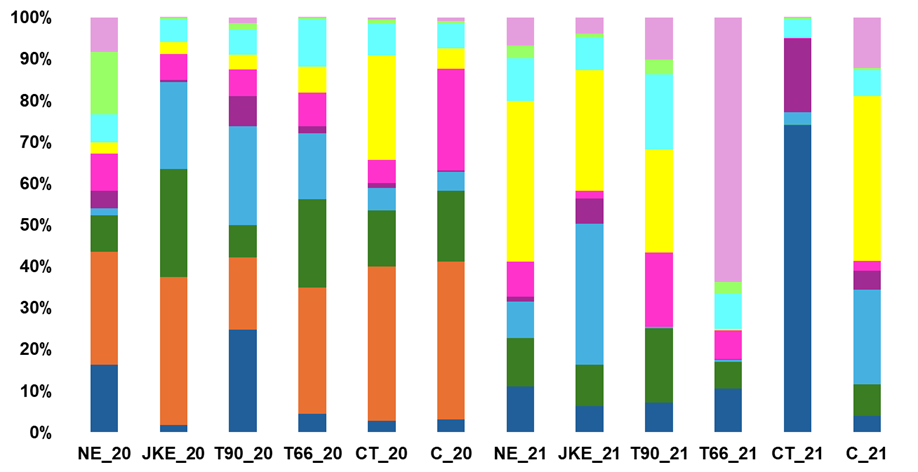


**Supplementary Figure 8.** Relative abundance of bacterial community (at family level) detected in cv. ‘Touriga Franca’ with six different foliar treatments, at harvest of 2020 and 2021. Only the top 10 bacterial families are presented. NE – Nettle extract; JKE – Japanese knotweed extract; T66 and T90 – Yeast extract; CT – Conventional treatment; C – Control.


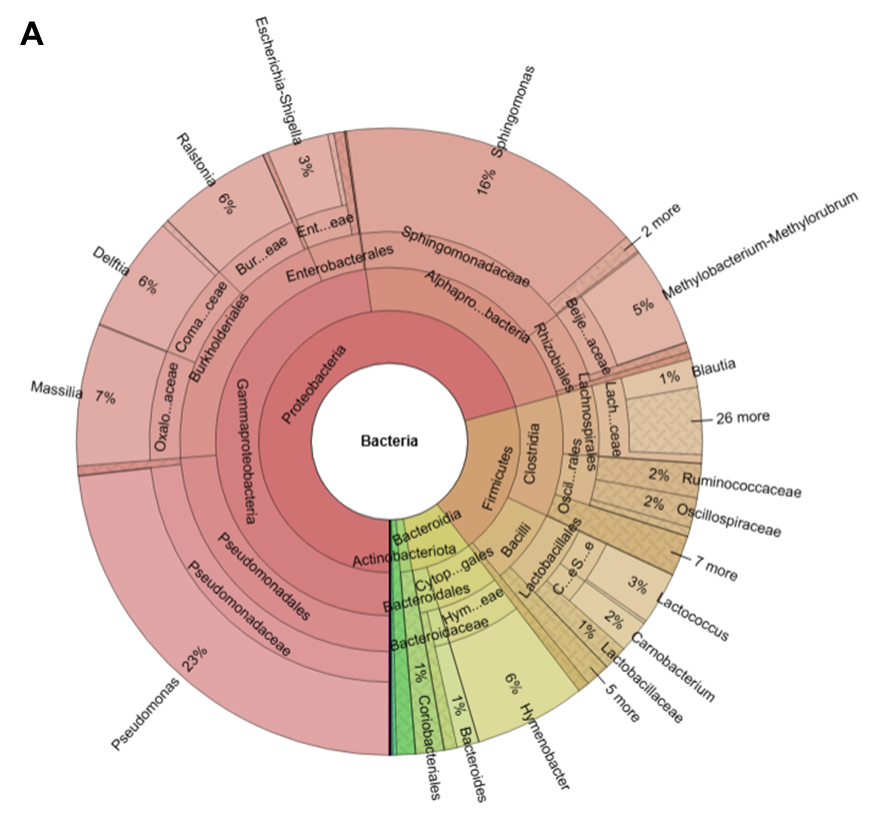


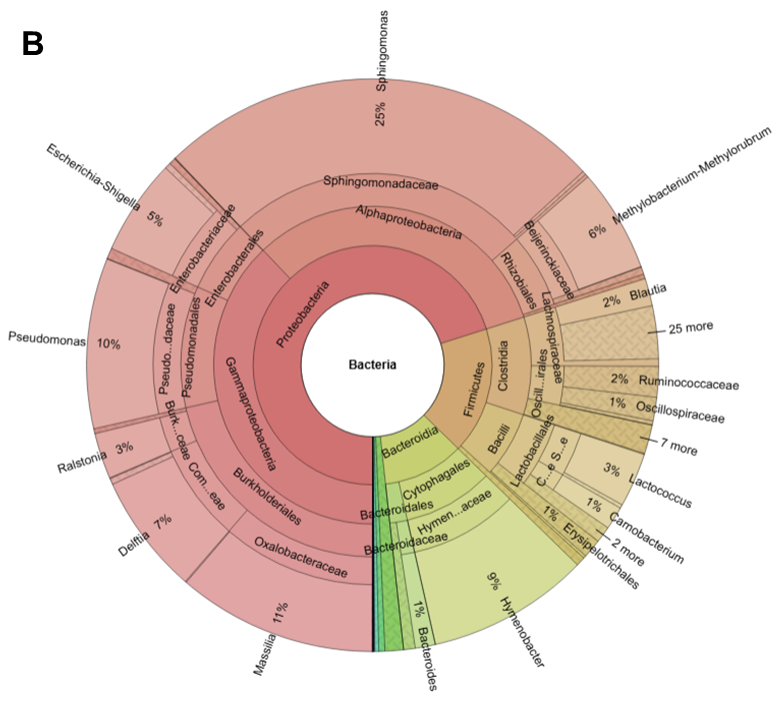


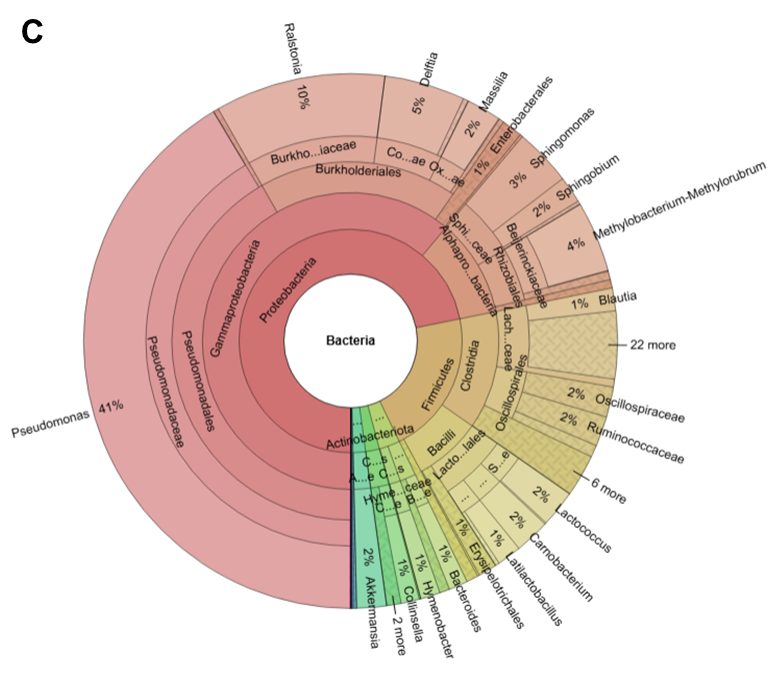


**Supplementary Figure 9.** Krona charts representing the relative abundance of the whole bacterial communities detected in both years (A), in 2020 (B) and in 2021 (C) in all six treatments.


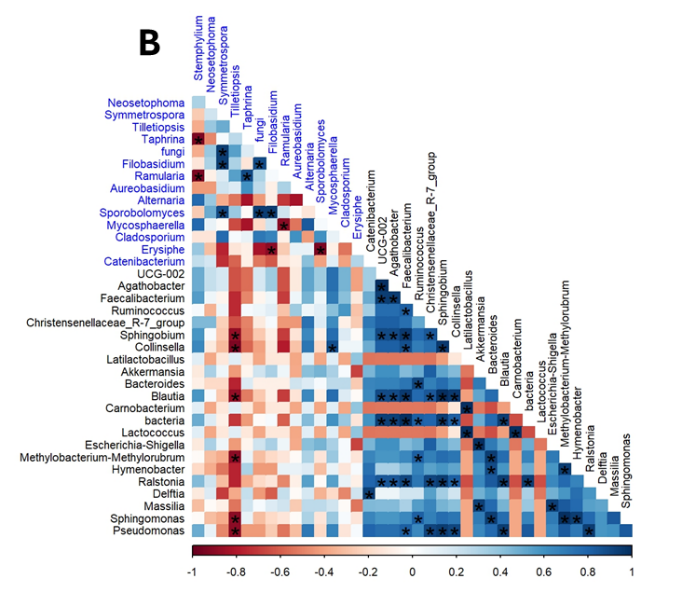

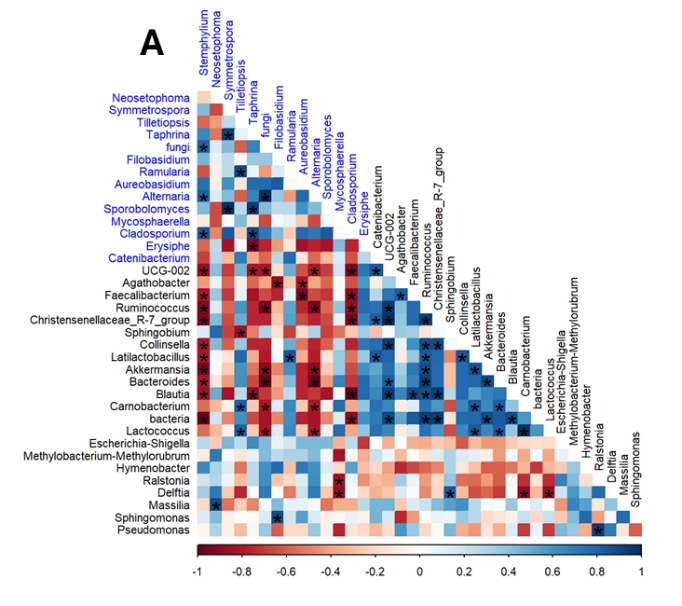

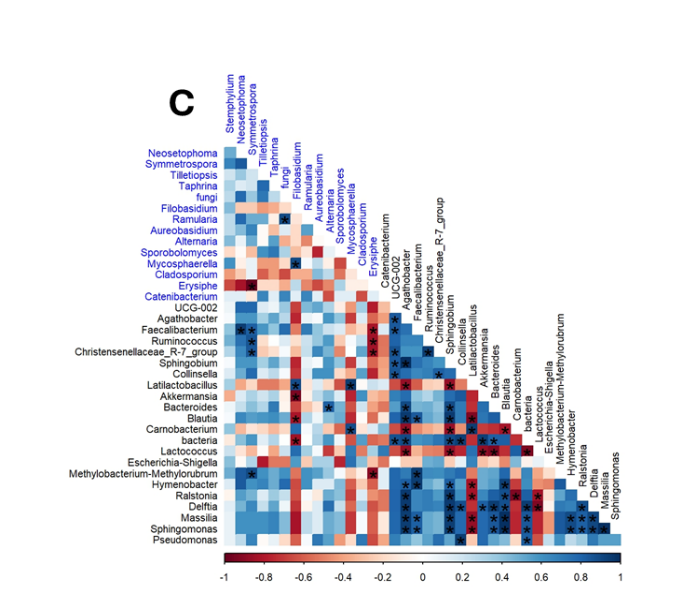

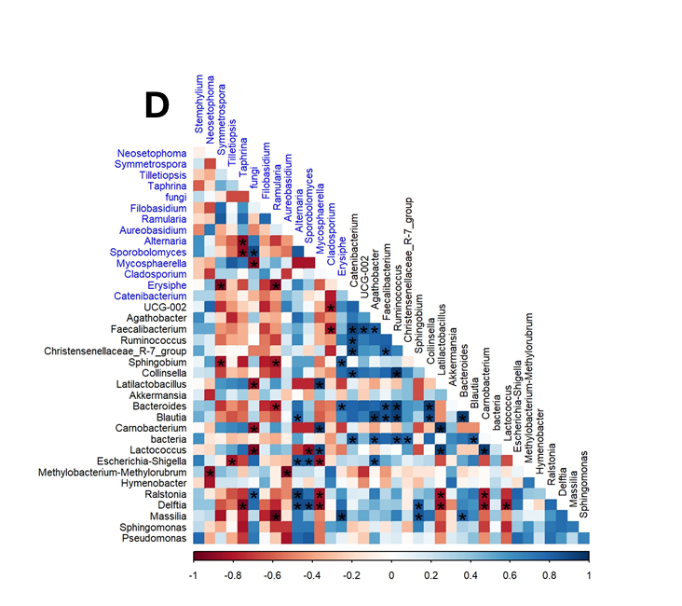

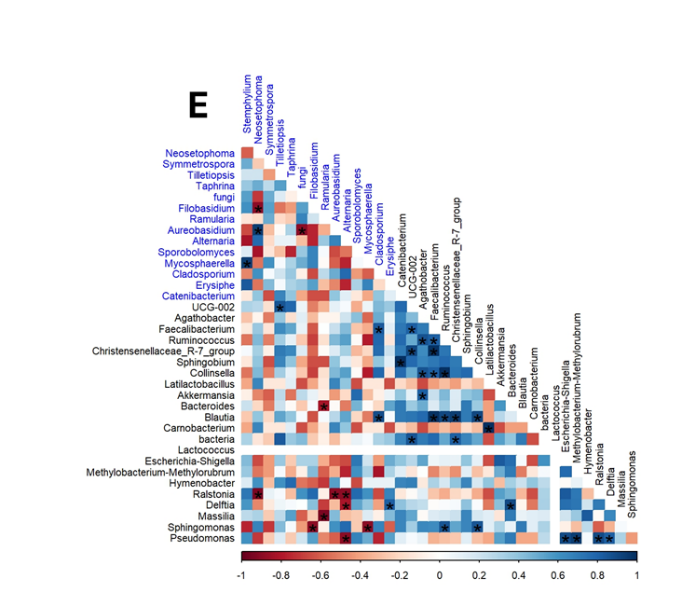

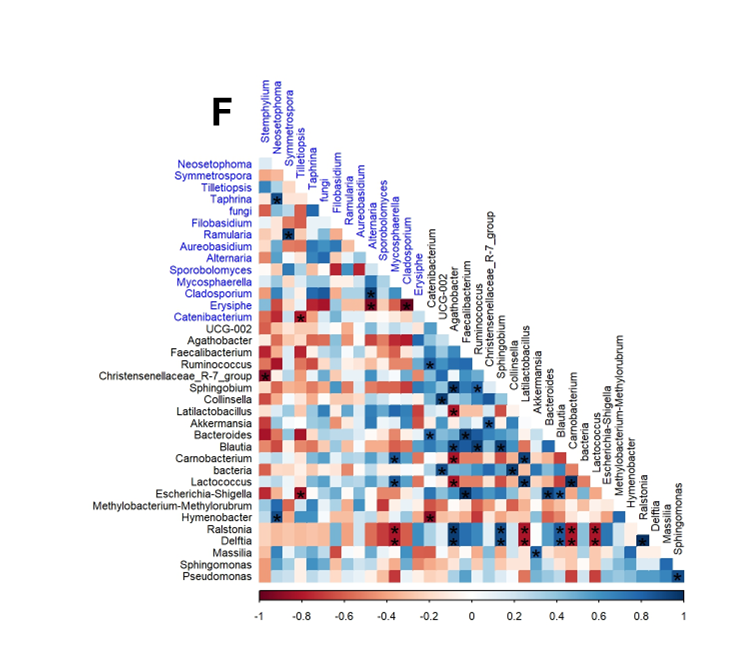


**Supplementary Figure 10.** Spearman correlation analysis between the relative abundances of bacterial and fungal genera of the overall community of the different treatments: Nettle extract (A); Japanese knotweed extract (B); T66 (C); T90 (D); Conventional treatment (E) and Control (F). Correlation coefficients are colored from dark red (negative correlation) to dark blue (positive correlation), with color intensity being proportional to the correlation coefficients. Bacterial and fungal genera names are colored in black and blue, respectively. Only genera representing more than 0.5% of the relative abundance of total reads in the complete dataset are represented. Statistically significant correlations are denoted with an asterisk (*p* < 0.05).
